# Characterization of Grape Cane Stilbenoids and Their Photo‐Degradation Products by HPLC‐DAD Coupled With Trapped Ion Mobility Spectrometry and High‐Resolution Tandem Mass Spectrometry

**DOI:** 10.1002/rcm.70074

**Published:** 2026-05-05

**Authors:** Paul Besrukow, Friederike A. Schnitker, Ralf Schweiggert, Christof B. Steingass

**Affiliations:** ^1^ Department of Beverage Research Geisenheim University Geisenheim Germany

**Keywords:** CCS, light, resveratrol, TIMS, viticulture

## Abstract

**Rationale:**

Bioactive (*E*)‐stilbenoids in vine extracts are prone to light‐induced transformation into the respective (*Z*)‐isomers and further degradation products, often resulting in a loss of bioactivity. We sought to study stilbenoid degradation beyond that of the well‐researched resveratrol due to the high diversity of stilbenoids in nature.

**Methods:**

Nine authentic, individual stilbenoids and a stilbenoid‐rich grape cane extract were irradiated with artificial sunlight and their photodegradation products analyzed by liquid chromatography coupled with diode array detection, electrospray ionization, trapped ion mobility spectrometry, and quadrupole time‐of‐flight high‐resolution tandem mass spectrometry (HPLC‐DAD‐ESI‐TIMS‐QTOF‐HR‐MS/MS).

**Results:**

Overall, a total of 45 different transformation products were identified in light‐exposed standard solutions and the grape cane extract. For the first time, collision cross section values of grape cane stilbenoids (154.8–286.4 Å^2^) and their degradation products (116.5–289.3 Å^2^) are reported. TIMS allowed the separation of isomeric and isobaric photodegradation products that would be challenging to differentiate when not resolved by chromatography and sharing similar HR‐MS/MS fragmentation patterns, e.g., certain trihydroxyphenanthrenediones.

**Conclusion:**

These results highlight the potential of combining liquid chromatography, TIMS, and HR‐MS/MS for these types of compounds to broaden the knowledge on photodegradation pathways of grapevine stilbenoids beyond resveratrol.

## 1 Introduction

Stilbenoids are phenolic compounds known for their diverse biological activities, including antioxidant and antimicrobial properties. They are widely considered as phytoalexins, playing a crucial role in plants' defense mechanisms against environmental and biotic stresses [1]. As such, they accrue in plant material including agricultural byproducts like grape cane, rendering them attractive targets for plant‐based developments of pharmaceutical, cosmetic and crop protection industries [2, 3]. Resveratrol as the most prominent stilbenoid is regarded as the precursor for more than 60 naturally occurring derivatives, resulting from modifications like glycosylation (e.g., piceid), hydroxylation (e.g., piceatannol) as well as di‐ (e.g., ɛ‐viniferin) and oligomerization (e.g., hopeaphenol) with different bioactive potentials [4]. Beyond this diversity, stilbenoids occur as (*E*)‐ and (*Z*)‐isomers. The (*E*)‐isomers have been generally found to be the more bioactive ones [5].

The *E/Z*‐isomerization of stilbenoids has been described to be facilitated under visible light or UV irradiation, being followed by further often only scarcely described degradation pathways. In this regard, (*E*)‐resveratrol is the most studied stilbenoid, being reported to initially isomerize into (*Z*)‐resveratrol. Further non‐stilbenoidic photodegradation products have been reported, such as 3,4′,5‐trihydroxy‐diphenylacetylene, 2,4,6‐phenanthrenetriol, 2‐(4‐hydroxyphenyl)‐5,6‐benzofuran‐dione, and aldehyde‐like transformation products [6, 7, 8]. Information on the photo‐induced transformation of further stilbenoids, however, is hitherto scarce.

Precise identification and quantification of stilbenoids are of great interest for improving our understanding of stilbenoids' structural diversity and the respective biological activity. To date, analytical techniques have mainly focused on high‐performance liquid chromatography (HPLC) combined with mass spectrometry (MS) [2]. However, the identification of isomeric compounds can be challenging due to the limited availability of reference standards. The recent advent of improved ion mobility spectrometry (IMS) techniques could be a valuable asset for analyzing complex (isomeric) mixtures of plant constituents like stilbenoids and their degradation products [9]. IMS facilitates the separation of isomeric compounds, thus bolstering confidence in their identification considering ion mobility values. A comparably novel IMS approach known as trapped IMS (TIMS) employs a tunnel mechanism to capture charged ions and segregate them based on their mobility values [10].

Our study followed two primary objectives. First, the transformation of nine distinct individual and authentic stilbenoids under artificial sunlight irradiation should be investigated in model solutions, being complemented by analyzing an irradiated stilbenoid‐rich extract of grape ( *Vitis vinifera* L.) cane. Second, we sought to assess the usefulness of trapped ion mobility spectrometry and to provide collision cross‐section (CCS) values for these stilbenoids and their photodegradation products, thereby offering an additional parameter for a more robust compound identification for those not having authentic reference compounds. Furthermore, the study explored the general feasibility of trapped ion mobility spectrometry (TIMS) in differentiating isomeric stilbenoids based on their ion mobility values.

## 2 Materials and Methods

### 2.1 Chemicals

Authentic standards (> 99% purity) of all (*E*)‐resveratrol, (*E*)‐piceatannol, (*E*)‐piceid, (*E*)‐ɛ‐viniferin, 4‐hydroxybenzaldehyde, 3,4‐ and 3,5‐dihydroxybenzaldehyde as well as 3,4‐ and 3,5‐dihydroxybenzoic acid were purchased from Extrasynthèse (Genay, France). (*Z*)‐resveratrol was obtained from Biomol (Hamburg, Germany). Ampelopsin A, hopeaphenol as well as r‐ and r2‐viniferin isolated from grape canes according to Macke et al. [11] were kindly provided by the research group of Prof. Dr. Peter Winterhalter (Institute of Food Chemistry, Technical University of Braunschweig, Germany). Furthermore, acetonitrile (Merck, Darmstadt, Germany) and LC–MS‐grade formic acid (ScienTest‐BioKEMIX, Leese, Germany) as well as ultrapure water (Elga Purelab, Celle, Germany) were used. (*Z*)‐isomers were produced by exposing the above‐mentioned authentic reference compounds being (*E*)‐isomers to irradiation emitted by the solar simulation system SOL500 (Hoenle, Gilching, Germany) as described before by our working group [12]. The below‐mentioned 10 mM sodium formate calibration solution was prepared by mixing 12.5 mL of water, 12.5 mL of isopropyl alcohol, 50 μL of formic acid (LC–MS grade, > 99%), and 250 μL of a 1 M aqueous sodium hydroxide solution.

### 2.2 Preparation of Grape Cane Extracts

Grape cane extracts were produced in accordance to Besrukow et al. [13] by chopping, drying and milling grape cane samples of *V. vinifera* L. cv. Pinot Noir to an approximate size of 1 mm. Extraction was performed with aqueous ethanol (80% vol., solid‐to‐solvent‐ratio 1:5, w/v) for 4 h at room temperature without light exposure in a technical scale. After ethanol evaporation until ca. 15% total solids concentration (Heidolph Hei‐VAP Industrial, Schwabach, Germany), the crude extract was spray dried (Büchi B‐290, Flawil, Switzerland) utilizing the carrier maltodextrin (30/70 carrier/extract ratio) and operating parameters of 150°C inlet air temperature, 80°C–82°C outlet temperature, 52 mL/min feed flow rate, and 5500 L/h air flow rate. The resulting powder was stored in the dark at 5°C until further usage.

### 2.3 Irradiation of Stilbenoids With Artificial Sunlight

Stilbenoid standards were dissolved individually in 5 mL of a mixture of water and acetonitrile (85/15, v/v) to obtain 0.1 mM solutions. The grape cane extract solution was produced in accordance to Besrukow et al. [12] by dispersing 250 mg of the above mentioned grape cane extract powder in 25 mL of the aforementioned mixture of water and acetonitrile. The stilbenoid concentrations in the powdery extract were previously reported [13]. The dissolved extract solution contained ca. 1.62, 0.45, 0.04, 0.64, 0.14, and 0.05 mM of (*E*)‐resveratrol, (*E*)‐piceatannol, (*E*)‐ampelopsin A, (*E*)‐ε‐viniferin, hopeaphenol and r‐viniferin, respectively. The 10 solutions (nine standard and one grape cane extract solution) were individually filtered through 0.2 μm glass fiber membranes (Chromafil Xtra, Macherney‐Nager, Dueren, Germany) into both brown and clear glass vials which were subsequently cap‐closed. While the brown glass vials were used for immediate HPLC analyses (see below), the clear glass vials underwent a 2‐h exposure to simulated sunlight with an illuminance of ca. 14.000 lx, emanating from the SOL500 solar simulation system (Hoenle, Gilching, Germany). The apparatus, equipped with a fan mechanism, maintained a consistent ambient temperature of 24°C–25°C throughout illumination. The distance between samples and light source was set at 1 m, which was positioned in a vertically oriented configuration directly above the samples. After light exposure, the samples in the clear glass vials were analyzed alongside the nonirradiated samples in the brown glass vials.

### 2.4 HPLC‐DAD‐ESI(−)‐TIMS‐QTOF‐HR‐MS/MS Analyses

Stilbenoid analyses were performed using a Bruker Elute UHPLC system (Bruker Daltonics, Bremen, Germany) equipped with a Hypersil Gold RP‐18 column (150 mm × 2.1 mm inner diameter, 3 μm particle size, Thermo Fisher Scientific, Dreieich, Germany). The mobile phase consisted of 0.1% (v/v) aqueous formic acid (A) and acetonitrile (B) used at a flow rate of 0.3 mL/min. A linear elution gradient of 21 min was run at 40°C under the following conditions: 0 min (15% B), 10 min (50% B), 11 min (50% B), 12 min (100% B), 16 min (100% B), 17 min (15% B), and 21 min (15% B). The column oven temperature was 40°C, the injection volume 3 μL. UV/vis absorption spectra were recorded applying a diode array detector (DAD) in the range of 200–600 nm. Detection wavelengths were 280, 306, and 320 nm.

The HPLC system was interfaced with a trapped ion mobility spectrometer (TIMS) and a quadrupole time‐of‐flight high‐resolution tandem mass spectrometer (QTOF‐HR‐MS/MS, *tims*TOF Pro 2, Bruker Daltonics) with an electrospray ionization (ESI) source operating in negative ion mode. Capillary voltage was 3200 V and nebulizer temperature 200°C. Nitrogen was the nebulizing and drying gas applied at 2.5 bar and 6 mL/min, respectively. HR‐MS and HR‐MS/MS spectra were recorded within a range of *m/z* 20–1300. Collision energy for collision induced dissociation (CID) was 20 and 50 eV. TIMS nitrogen gas was produced using a membrane‐based generator with an exit pressure of 7 bar. Ion mobilities were measured from 0.45 to 1.45 Vs/cm^2^ with a ramp time of 100.0 ms. Ion charge control (ICC) was set to 2 million counts. Agilent ESI‐L Low Concentration Tuning Mix and the abovementioned 10 mM sodium formate calibrant solution were used for initial calibration of the TIMS (linear, search range: 0.01 1/K_0_, intensity threshold: 3000 counts) and the MS (high precision calibration [HPC], search range: *m/z* 0.05, intensity threshold: 5000 counts), respectively, prior to each analytical series. At the beginning of each analytical run, 20 μL of a mixture (3:1, v/v) of the Tuning Mix and the sodium formate calibrant were injected for internal calibration during data processing. Default mobility values (*m/z* 301.9981: 0.6690 Vs/cm^2^, *m/z* 601.9790: 0.8824 Vs/cm^2^, *m/z* 1033.9881: 1.2582 Vs/cm^2^) from the “Tuning Mix ESI‐TOF CCS compendium (ESI)” were used [14]. The HPLC was operated with Bruker Compass HyStar version 6.0.30.0, while Bruker otofControl version 6.0.115 managed the MS system. To evaluate the reproducibility of the CCS measurements, all used stilbenoid parent compounds and six selected degradation products, for all of which reference standards had been available, were analyzed in triplicate (*n* = 3).

### 2.5 Data Processing

Data were processed with Bruker DataAnalysis 6.1 software. Ion mobilograms were created by isolating extracted ion mobilograms (EIMs) of the respective deprotonated molecules ([M–H]^−^) in MS1 spectra following smoothing using a single cycle Savitzky Golay algorithm and 0.01 [V*s/cm^2^] as smoothing widths [15]. CCS values were calculated based on dominant *m/z* (singly charged ion species). Statistically significant differences between the means of isomeric compounds (*p* < 0.05, *n* = 3) were assessed using a two‐sample *t*‐test performed in R (RStudio version 2026.01.1).

## 3 Results and Discussion

Stilbenoid identification was based on their retention time (*t*_R_), elution order, UV absorption spectra, *m/z* of the negatively charged deprotonated molecules [M–H]^−^, and ESI(−)‐HR‐MS/MS mass fragmentations that were also compared to those from nine authentic reference standards and literature (cited in the following). Additionally, ion mobility and CCS values were investigated to provide a further parameter for differentiation. Table 1 lists the nine authentic reference standards as parent compounds categorized into monomeric, dimeric, and tetrameric stilbenoids including the respective observed degradation products after 2 h exposure to high intensity artificial sunlight.

**TABLE 1:** HPLC‐DAD‐ESI(−)‐TIMS*‐*QTOF‐HR‐MS/MS data of nine stilbenoids (parent compound, bold) including proposed photo‐degradation products generated illuminating standard solutions.

#	Parent compound proposed photodegradation product	*t*_R_ (min)	λ_max_ (nm)	Exp. mass [M–H]^−^ (*m/z*)	Calc. mass [M–H]^−^ (*m/z*)	Error (ppm)	Sum formula	ESI(−)‐HR‐MS/MS (*m/z* (% base peak intensity))	^TIMS^CCS_N2_ (Å^2^) ± CV (%)	Mobility 1/K_0_ (Vs/cm^2^)
	Monomers									
A0	(*E*)‐resveratrol^§^	5.9	305, 320	227.0715	227.0714	–0.7	C_14_H_11_O_3_^−^	185.0608 (16), 143.0506 (14), 115.0551 (7), 41.0041 (9)	157.5 ± 0.08	0.742
A1	4‐hydroxybenzaldehyde^§^	3.9	280	121.0296	121.0295	−0.9	C_7_H_5_O_2_^−^	92.0269 (29), 41.0045 (7)	116.7	0.525
A2	Hydroxyphenylethenyl Butenedioic acid	4.0	280	233.0460	233.0455	−2.7	C_12_H_9_O_5_^−^	189.0561 (17), 145.0665 (100)	149.5	0.705
A3	Hydroxyphenylethinyl butenedioic acid	4.6	280	231.0302	231.0299	−1.3	C_12_H_7_O_5_^−^	187.0401 (37), 143.0504 (100)	145.2	0.685
A4	(*E*)‐resveratrol^§^	5.9	305, 321	227.0715	227.0714	−0.7	C_14_H_11_O_3_^−^	185.0611 (21), 143.0521 (14), 115.0551 (4), 41.0050 (3)	157.5	0.742
A5	Unknown 1	6.9	274	211.0402	211.0401	−0.8	C_13_H_7_O_3_^−^	167.0506 (6), 141.0338 (13)	141.0	0.662
A6	Dihydroxyphenanthrenedi‐one	7.3	280	239.0352	239.0350	−1.1	C_14_H_7_O_4_^−^	211.0406 (83), 167.0490 (8), 143.0510 (13)	149.6	0.707
A7	(*E*)‐ɛ‐viniferin^§^	7.4	320	453.1344	453.1345	−0.3	C_28_H_21_O_6_^−^	365.0895 (72), 328.1716 (55), 257.9959 (48), 115.0530 (77)	206.5	1.001
A8	Hydroxyphenylethinyl butenedioic acid anhydride	8.8	270	213.0195	213.0193	−0.9	C_12_H_5_O_4_^−^	169.0298 (13), 141.0349 (100)	136.6	0.641
B0	(*Z*)‐resveratrol^§^	7.0	285	227.0715	227.0714	−0.6	C_14_H_11_O_3_^−^	185.0614 (19), 143.0505 (17), 115.0554 (9), 41.0041 (12)	154.8 ± 0.01	0.730
B1	3,4‐ & 3,5‐dihydroxybenz‐aldehyde^§^	3.1	nd	137.0243	137.0244	0.8	C_7_H_5_O_3_^−^	108.0226 (16), 93.0365 (6), 41.0051 (9)	118.2	0.538
B2	4‐hydroxybenzaldehyde^§^	3.9	nd	121.0291	121.0295	3.5	C_7_H_5_O_2_^−^	92.0272 (38), 41.0058 (2)	116.5	0.524
B3	(*E*)‐piceatannol^§^ ^,^ ^b^	4.7	320	243.0665	243.0663	−0.2	C_14_H_11_O_4_^−^	201.0576 (23), 159.0469 (6)	159.6	0.755
B4	Trihydroxyphenanthrene‐dione 1	5.0	nd	255.0301	255.0299	−0.7	C_14_H_7_O_5_^−^	227.0364 (14), 183.0438 (8)	147.1	0.697
B5	Trihydroxyphenanthrene‐dione 2	5.6	nd	255.0303	255.0299	−1.4	C_14_H_7_O_5_^−^	227.0334 (13), 183.0514 (8)	147.1	0.697
B6	(*Z*)‐piceatannol	5.7	nd	243.0665	243.0553	−1.0	C_14_H_11_O_4_^−^	201.0563 (17), 159.0452 (19)	156.9	0.742
C0	(*E*)‐piceatannol^§^	4.7	323	243.0661	243.0663	0.9	C_14_H_11_O_4_^−^	225.0565 (4), 201.0561 (11), 159.0456 (12), 131.0514 (4), 41.0038 (6)	159.5 ± 0.05	0.755
C1	Dihydroxybenzoic acid	2.4	254	153.0193	153.0193	−0.9	C_7_H_5_O_4_^−^	109.0295 (100)	120.8	0.554
C2	3,4‐dihydroxybenzaldehyde^§^	3.0	260	137.0245	137.0244	−0.5	C_7_H_5_O_3_^−^	108.0214 (11), 41.0039 (4)	118.1	0.538
C3	(*E*)‐piceatannol^§^	4.7	320	243.0665	243.0663	−0.2	C_14_H_11_O_4_^−^	225.0543 (3), 201.0576 (23), 159.0469 (6), 131.0478 (2), 41.0041 (5)	159.6	0.755
C4	Tetrahydroxyphenanthrene	5.0	254	241.0511	241.0506	−2.0	C_14_H_9_O_4_^−^	213.0561 (9), 197.0604 (4), 183.0453 (4),169.0303 (6)	148.6	0.702
C5	Trihydroxyphenanthrene‐dione 2	5.6	265	255.0304	255.0299	−1.8	C_14_H_7_O_5_^−^	227.0362 (10), 183.0557 (6)	147.0	0.697
C6	Tetrahydroxyphenanthrene‐dione	5.6	265	271.0248	271.0248	0.1	C_14_H_7_O_6_^−^	243.0303 (11), 227.0372 (6)	150.0	0.713
C7	(*Z*)‐piceatannol	5.8	nd	243.0667	243.0553	−1.7	C_14_H_11_O_4_^−^	225.0550 (4), 201.0557 (13), 159.0451 (15), 131.0499 (4), 41.0041 (5)	156.9	0.742
C8	Piceatannol‐dimer 1	5.9	nd	485.1239	485.1242	0.6	C_28_H_21_O_8_^−^	375.0874 (9), 240.0436 (4), 109.0297 (10)	207.6	1.008
C9	Piceatannol‐dimer α‐aldehyde	7.3	265	379.0827	379.0823	−0.9	C_21_H_15_O_7_^−^	243.0665 (5), 137.0245 (5)	195.5	0.944
C10	Dihydroxyphenylethinyl butenedioic acid anhydride	7.5	264	229.0145	229.0142	−0.9	C_12_H_5_O_5_^−^	185.0246 (5), 157.0297 (58)	138.2	0.651
C11	Piceatannol‐dimer 2	7.7	nd	485.1240	485.1242	0.4	C_28_H_21_O_8_^−^	243.0671 (67), 241.0510 (78), 197.0614 (12)	211.1	1.025
C12	Oxidized piceatannol‐dimer 1	8.2	264, 368	483.1085	483.1085	0.0	C_28_H_19_O_8_^−^	374.4072 (98), 239.0355 (59)	228.2	1.108
C13	Piceatannol‐dimer 3	8.8	365	485.1246	485.1242	−0.9	C_28_H_21_O_8_^−^	243.0665 (88), 241.0507 (100), 197.0607 (23)	211.0	1.025
C14	Oxidized piceatannol‐dimer 2	9.4	260	483.1087	483.1085	−0.3	C_28_H_19_O_8_^−^	243.0642 (76), 241.0468 (70), 197.0614 (17)	223.8	1.087
D0	(*E*)‐piceid^§^	4.1	306, 320	389.1242	389.1242	−0.1	C_20_H_21_O_8_^−^	227.0728 (100), 185.0611 (8), 143.0488 (11)	182.1 ± 0.05	0.878
D1	Piceid formic acid adduct 1^b^			435.1303	435.1297	−1.5	C_20_H_23_O_10_^−^	389.1245 (47), 227.0711 (100), 185.0611 (4)	192.1	0.927
D2	Piceid formic acid adduct 2^b^			435.1297	435.1297	−0.2	C_20_H_23_O_10_^−^	389.1245 (66), 227.0712 (100), 185.0613 (6), 143.0502 (5)	208.4	1.006
D3	Dihydroxybenzoic acid‐*O*‐glucose 1	2.2	nd	315.0726	315.0722	−1.5	C_13_H_15_O_9_^−^	153.0197 (100), 109.0296 (28), 67.0194 (30), 65.0402 (22)	167.6	0.802
D4	Dihydroxybenzoic acid‐*O*‐glucose 2	2.2	nd	315.0726	315.0722	−1.5	C_13_H_15_O_9_^−^	153.0183 (100), 109.0296 (35), 67.0198 (16), 65.0410 (35)	175.2	0.838
D5	Hydroxybenzoic acid‐*O*‐glucose 1/dihydroxybenz‐aldehyde‐*O*‐glucose 1	2.3	nd	299.0775	299.0772	−1.0	C_13_H_15_O_8_^−^	137.0246 (100), 93.0346 (39)	171.7	0.820
D6	4‐hydroxybenzaldehyde^§^	3.9	282	121.0296	121.0295	−0.1	C_7_H_5_O_2_^−^	92.0265 (41), 41.0042 (4)	116.6	0.528
D7	Hydroxybenzoic acid‐*O*‐glucose 2/dihydroxybenz‐aldehyde‐*O*‐glucose 2	4.7	274	299.0776	299.0772	−1.1	C_13_H_15_O_8_^−^	137.0243 (100), 93.0350 (33)	169.2	0.808
D8	Unknown 2	5.0	285	401.0881	401.0878	−0.7	C_20_H_17_O_9_^−^	239.0353 (10), 210.0341 (4), 182.0381 (3), 166.0429 (18)	194.2	0.938
D9	Unknown 3	5.7	283	399.0724	399.0350	−0.5	C_20_H_15_O_9_^−^	237.0193 (3), 166.0413 (6), 153.0343 (4)	190.8	0.921
D10	Dihydroxyphenanthrenedione	7.2	nd	239.0352	239.0350	−0.6	C_14_H_7_O_4_^−^	211.0407 (84), 167.0523 (15), 143.0526 (14)	149.7	0.707
D11	Hydroxyphenylethinyl butenedioic acid anhydride	8.7	270	213.0195	213.0193	−0.6	C_12_H_5_O_4_^−^	169.0294 (12), 141.0350 (100)	136.5	0.641
	Dimers									
E0	(*E*)‐ampelopsin A^§^	4.4	281	469.1296	469.1293	−0.7	C_28_H_21_O_7_^−^	451.1136 (100), 375.0880 (52), 363.0853 (17), 121.0389 (80)	202.6 ± 0.08	0.982
	‐ Formic acid adduct			515.1344	515.1348	0.7	C_29_H_23_O_9_^−^	469.1298 (100)	213.8	1.037
E1	Dihydroxybenzoic acid	2.4	250, 300	153.0195	153.0193	−1.2	C_7_H_5_O_4_^−^	109.0295 (8)	121.0	0.555
E2	4‐hydroxybenzoic acid^§^	3.0	254	137.0244	137.0244	0.0	C_7_H_5_O_3_^−^	108.0229 (16), 93.0344 (29), 91.0187 (15), 41.0039 (5)	118.3	0.539
E3	4‐hydroxybenzaldehyde^§^	3.8	282	121.0298	121.0295	−0.3	C_7_H_5_O_2_^−^	92.0270 (31), 41.0041 (5)	116.5	0.525
E4	(*E*)‐ampelopsin A	4.5	281	469.1296	469.1293	−0.7	C_28_H_21_O_7_^−^	451.1136 (89), 375.0887 (48), 363.0815 (42), 121.0296 (100)	202.5	0.982
F0	(*E*)‐ɛ‐viniferin^**§**^	7.4	323	453.1342	453.1344	0.3	C_28_H_21_O_6_^−^	347.0938 (7), 147.0456 (6)	206.1 ± 0.02	1.000
	(*E*)‐ɛ‐viniferin formic acid adduct^b^			499.1395	499.1398	0.6	C_29_H_23_O_8_^−^	453.1348 (100)	219.6	1.064
F1	Dihydroxybenzaldehyde	3.1	266	137.0247	137.0244	−1.7	C_7_H_5_O_3_^−^	93.0338 (92), 41.0040 (33)	120.5	0.548
F2	4‐hydroxybenzoic acid^§^	3.1	254	137.0246	137.0244	−1.5	C_7_H_5_O_3_^−^	108.0213 (12), 93.0341 (18), 41.0040 (6)	118.4	0.544
F3	4‐hydroxybenzaldehyde^§^	3.8	282	121.0296	121.0295	−1.2	C_7_H_5_O_2_^−^	92.0270 (29), 41.040 (6)	116.7	0.525
F4	α acid	5.1	nd	379.0825	379.0823	−0.5	C_21_H_15_O_7_^−^	335.0929 (37), 273.0414 (52), 272.0342 (46)	199.5	0.961
F5	Pentahydroxyphenanthrene	5.8	nd	257.0460	257.0455	−1.7	C_14_H_9_O_5_^−^	121.0297 (8)	160.4	0.760
F6	α aldehyde of F0	5.8	nd	363.0874	363.0874	0.4	C_21_H_15_O_6_^−^	—	193.1	0.929
F7	Dihydroxyphenanthrenedione	7.3	282	239.0354	239.0350	−1.5	C_14_H_7_O_4_^−^	211.0403 (89), 167.0499 (13), 143.0520 (6)	149.8	0.708
F8	Trihydroxyphenanthrene‐dione 3	7.3	282	255.0300	255.0299	−0.5	C_14_H_7_O_5_^−^	227.0350 (19), 211.0394 (87), 167.0495 (92)	148.9	0.706
F9	Trihydroxyphenanthrene‐dione 4	7.3	282	255.0300	255.0299	−0.5	C_14_H_7_O_5_^−^	227.0350 (19), 211.0394 (87), 167.0495 (92)	153.5	0.728
F10	Hydroxyphenylethinyl butenedioic acid anhydride	8.8	270	213.0193	213.0193	0.1	C_12_H_5_O_4_^−^	169.0292 (9), 141.0348 (100)	136.7	0.642
F11	ɛ‐viniferin dihydroxy‐phenanthrenedione	9.0	nd	481.0930	481.0929	−0.1	C_28_H_17_O_8_^−^	453.0985 (11), 437.1057 (100), 409.1103 (33), 331.0590 (42)	nd	nd
	‐ Formic acid adduct			527.0981	527.0984	−0.5	C_29_H_19_O_10_^−^	481.0951 (21), 454.0990 (21), 453.0960 (59), 437.1048 (100), 409.1087 (46), 331.0594 (21)	222.2	1.081
	Tetramers									
G0	Hopeaphenol^§^	6.6	280	905.2610	905.2604	−0.7	C_56_H_41_O_12_^−^	—	286.4 ± 0.15	1.411
G1	Dihydroxybenzoic acid^§^	2.4	255, 300	153.0192	153.0193	−1.0	C_7_H_5_O_4_^−^	109.0295 (100)	121.6	0.558
G2	4‐hydroxybenzoic acid^§^ ^,^ ^b^	3.1	251	137.0244	137.0244	0.3	C_7_H_5_O_3_^−^	108.0225 (7), 93.0345 (42), 91.0190 (8) 41.0038 (10)	118.3	0.538
G3	4‐hydroxybenzaldehyde^§^	3.8	284	121.0296	121.0295	−1.2	C_7_H_5_O_2_^−^	92.0266 (22), 41.0408 (6)	116.5	0.524
G4	(*E*)‐ampelopsin A	4.4	281	469.1281	469.1293	−1.3	C_28_H_21_O_7_^−^	451.1196 (31), 375.1218 (4), 363.0878 (3), 121.0298 (100)	203.0	0.985
	‐ Formic acid adduct			515.1348	515.1348	−0.1	C_29_H_23_O_9_^−^	469.1293 (100)	213.2	1.037
G5	Hopeaphenol^§^	6.6	280	905.2610	905.2604	−0.7	C_56_H_41_O_12_^−^	—	286.4	1.408
G6	Unknown 4	6.8	283	451.1189	451.1187	−0.5	C_28_H_19_O_6_^−^	93.0352 (5)	nd	nd
	‐ Formic acid adduct			497.1241	497.1242	0.1	C_29_H_21_O_8_^−^	451.1187 (100)	208.9^c^	1.015^c^
H0	(*E*)‐r2‐viniferin^§^	7.9	284, 323	905.2610	905.2604	−0.7	C_56_H_41_O_12_^−^	811.2193 (1)	285.1 ± 0.13	1.402
H1	Dihydroxybenzoic acid^§^	2.4	250, 300	153.0194	153.0193	−0.3	C_7_H_5_O_4_^−^	109.0308 (35)	121.1	0.556
H2	3,5‐dihydroxybenzaldehyde^§^	3.0	268, 327	137.0244	137.0244	0.4	C_7_H_5_O_3_^−^	93.0344 (3), 41.0038 (14)	120.2	0.547
H3	4‐hydroxybenzoic acid^§^	3.1	255	137.0245	137.0244	−0.5	C_7_H_5_O_3_^−^	108.0207 (8), 41.0039 (14)	118.3	0.538
H4	4‐hydroxybenzaldehyde^§^	3.8	283	121.0297	121.0295	−1.6	C_7_H_5_O_2_^−^	92.0269 (32), 41.0439 (14)	116.6	0.525
H5	α acid of H0	5.1	nd	379.0826	379.0823	−0.5	C_21_H_15_O_7_^−^	335.0917 (44), 273.0399 (51), 272.0320 (34), 93.0345 (9), 41.0043 (5)	199.4	0.961
H6	α' acid 1 of H0	5.4	226, 280	589.1505	589.1504	−0.1	C_35_H_25_O_9_^−^	545.1606 (4), 495.1119 (2), 483.1066 (1), 439.1222 (1), 137.0252 (1)	236.5	1.154
H7	α aldehyde of H0	5.8	nd	363.0878	363.0874	−1.0	C_21_H_15_O_6_^−^	345.0777 (8), 335.0929 (4), 334.0867 (2), 93.0346 (9), 41.0052 (3)	192.8	0.928
H8	α' aldehyde‐like of H0 (1)	6.3	227, 280	573.1558	573.1555	−0.5	C_35_H_25_O_8_^−^	479.1124 (2), 121.0297 (4)	234.4	1.143
H9	(*Z*)‐r2‐viniferin	7.6	282	905.2600	905.2604	0.3	C_56_H_41_O_12_^−^	811.2145 (2)	289.3	1.423
H10	(*E*)‐r2‐viniferin^§^	7.9	284, 326	905.2602	905.2604	0.2	C_56_H_41_O_12_^−^	811.2172 (6)	287.9	1.416
H11	α' aldehyde‐like of H0 (2)	8.1	227, 282	573.1550	573.1555	0.8	C_35_H_25_O_8_^−^	121.0292 (7), 93.0337 (3)	234.8	1.145
I0	(*E*)‐r‐viniferin^§^	9.1	323	905.2607	905.2604	−0.4	C_56_H_41_O_12_^−^	—	285.2 ± 0.36	1.403
I1	Dihydroxybenzoic acid^§^	2.4	250, 300	153.0192	153.0193	0.7	C_7_H_5_O_4_^−^	109.0295 (100)	121.0	0.556
I2	3,5‐dihydroxybenzaldehyde^§^	3.0	268, 327	137.0245	137.0244	−0.5	C_7_H_5_O_3_^−^	93.0351 (31), 41.0046 (36)	120.4	0.548
I3	4‐hydroxybenzoic acid^§^	3.1	255	137.0245	137.0244	−0.6	C_7_H_5_O_3_^−^	108.0221 (10), 93.0354 (12)	118.2	0.538
I4	4‐hydroxybenzaldehyde^§^	3.8	284	121.0298	121.0295	−2.4	C_7_H_5_O_2_^−^	92.0269 (32), 41.0439 (5)	116.6	0.525
I5	α acid of I0	5.1	nd	379.0825	379.0823	−0.5	C_21_H_15_O_7_^−^	335.0929 (37), 273.0414 (52), 272.0342 (46), 41.0042 (14)	199.5	0.961
I6	α aldehyde of I0	5.8	nd	363.0878	363.0874	−1.0	C_21_H_15_O_6_^−^	345.0777 (8), 335.0929 (4), 334.0867 (2)	192.8	0.928
I7	(*E*)‐resveratrol^§^ ^,^ ^b^	5.8	305, 321	227.0715	227.0714	−0.7	C_14_H_11_O_3_^−^	185.0611 (21), 143.0521 (14)	157.7	0.743
I8	(*Z*)‐resveratrol^§^	7.0	282	227.0719	227.0714	−1.6	C_14_H_11_O_3_^−^	185.0614 (19), 143.0505 (17)	155.2	0.731
I9	α' acid of I0	7.2	nd	589.1509	589.1504	−0.8	C_35_H_25_O_9_^−^	545.1649 (29), 483.1114 (12), 451.1171 (10), 439.1163 (22), 121.0285 (19), 93.0350 (14)	237.3	1.158
I10	α' aldehyde of H0	8.1	227, 282	573.1560	573.1555	−0.8	C_35_H_25_O_8_^−^	—	234.0	1.141

Abbreviations: CCS, collision cross section; CV, coefficient of variation (*n* = 3); nd, not determined; *t*_R_, retention time; λ_max_, UV/Vis absorption maxima.

^a^ CCS and mobility were tentatively assigned.

^b^ Present in the untreated standard.

^§^ Confirmed by an authentic external standard.

### 3.1 Degradation of Monomeric Stilbenoids

#### 3.1.1 (*E*)‐Resveratrol

Chemical ionization dissociation (CID) of (*E*)‐resveratrol (Table 1, #A0, [M–H]^−^ at *m/z* 227.0715) yielded distinctive fragment ions at *m/z* 185.0608 ([M–H–C_2_H_2_O]^−^), 143.0506 ([M–H–C_2_H_2_O–C_2_H_2_O]^−^), and 115.0551 ([M–H–C_2_H_2_O–C_2_H_2_O–CO]^−^), thus following the fragmentation pattern previously reported for stilbene monomers [16]. Hereby, the eliminated C_2_H_2_O unit may represent ketene (H_2_C=C=O, calc. 42.0106 amu). Notably, the fragment ion at *m/z* 41.0041 may be attributed to a deprotonated species of either ketene and/or its tautomer ethynol (C_2_HO^−^, calc. *m/z* 41.0033), being detected in the HR‐MS/MS spectra of most stilbenoids and, notably, also as the base peak of 3,5‐dihydroxybenzoic acid (cf. Supporting Information).

**FIGURE 1:**
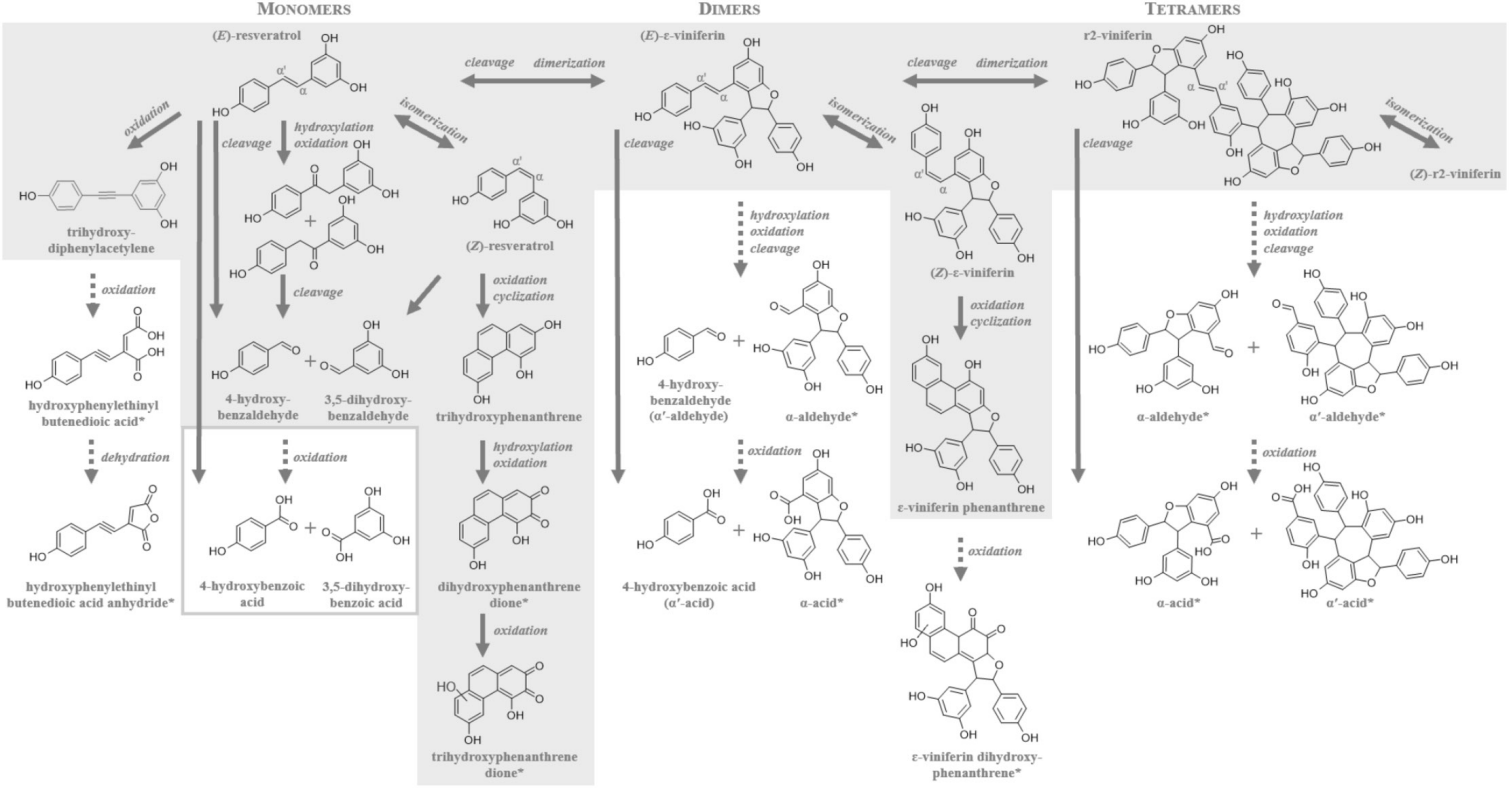
Proposed photodegradation products of mono‐, di‐ and tetrameric stilbenoids, exemplary for (*E*)‐resveratrol, (*E*)‐ε‐viniferin, and (*E*)‐r2‐viniferin, respectively. Gray area indicates stilbenoid photodegradation products previously reported in literature [6, 7, 17]. Structures in gray boxes have been previously reported to result from chemical oxidation of resveratrol [18]. Tentative structures are marked with an asterisk (*).

The photodegradation under artificial sunlight exposure yielded several distinct products, confirming multiple transformation pathways that had been suggested earlier (cf., Figure 1). Firstly, the observed mass at *m/z* 453.1344 ([M–H]^−^, Table 1, #A7) suggested the presence of a dimeric stilbenoid. As further based on a comparison of its *t*_R_ and CCS value with those of an authentic standard, this compound would tentatively be (*E*)‐ɛ‐viniferin. A light‐induced dimerization of (*E*)‐resveratrol leading to such dimers has previously been reported in the literature [19]. Notably, (*E*)‐resveratrol expectedly had a significantly smaller CCS value (157.5 Å^2^, CV 0.08%) than its related dimer (206.5 Å^2^). Secondly, dihydroxyphenanthrenedione (Table 1, #A6, [M–H]^−^ at *m/z* 239.0352) was tentatively identified in the irradiated sample according to its UV and mass spectral data (characteristic fragment ions from eliminations of CO and CO_2_), being in agreement with those reported by Rodríguez‐Cabo et al. [7] The latter authors reported that (*E*)‐resveratrol underwent a cascade of reactions including isomerization, cyclization, hydroxylation and oxidation into diverse phenanthrene‐ and diquinone‐like transformation products.

Thirdly, hydroxyphenylethenyl butenedioic acid (Table 1, #A2, [M–H]^−^ at *m/z* 233.0460 equaling C_12_H_9_O_5_^−^) was tentatively assigned by its sum formula and characteristic HR‐MS/MS fragment ions at *m/z* 189.0561 and 145.0665 ([M–H–44]^−^ and [M–H–44–44]^−^, resp.), possibly resulting from decarboxylations of the two carboxyl groups. The UV absorption maximum was as expected at 280 nm. The same approach was applied for tentative identification of hydroxyphenylethinyl butenedioic acid (Table 1, #A3, [M–H]^−^ at *m/z* 231.0302). Dore et al. [20] had suggested that phenol and resorcinol were degraded into the butenedioic acids maleic and fumaric acid by a ring‐opening mechanism, which was confirmed by other research groups [21, 22]. Given that (*E*)‐resveratrol incorporates a resorcinol bound to a hydroxyphenyl moiety via an ethenyl‐bridge, such a degradation mechanism may also apply here, bringing forth hydroxyphenylethenyl butenedioic acid (#A2), which, in turn, may be further oxidized to its triple‐bond derivative hydroxyphenylethinyl butenedioic acid (#A3), exhibiting an analogous fragmentation pattern. Likely, an anhydration of compound #A2 would then give rise to hydroxyphenylethinyl butenedioic acid anhydride (Table 1, #A8, [M–H]^−^ at *m/z* 213.0195), as tentatively identified based on its proposed molecular formula (C_12_H_5_O_4_^−^) and key fragment ions [M–H–44]^−^ and [M–H–44–28]^−^, representing the loss of CO_2_ and CO, respectively. To our knowledge, these compounds postulated here have not been reported as resveratrol photodegradation products. However, the structures tentatively proposed herein should be substantiated in continuative studies.

Earlier, Rodríguez‐Cabo et al. [7] have accentuated the photodegradation of resveratrol into “aldehyde like transformation products” via hydroxylation, oxidation and cleavage. In the irradiated (*E*)‐resveratrol sample, we unambiguously detected 4‐hydroxybenzaldehyde (Table 1, #A1, [M–H]^−^ at *m/z* 121.0296) as identified by comparing its UV and mass spectra to those of an authentic reference compound (cf. Supporting Information). Notaby, the even‐numbered product ions detected at *m/z* 92.0272 may resemble dehydrophenoxide radical anions (C_6_H_4_O^•–^) resulting from the radical loss of hydrogen (H^•^) following elimination of carbon monoxide (CO), equaling the elimination of CHO^•^ from the aldehyde‐group and being distinctive for *p*‐hydroxybenzaldehyde under ESI conditions [23, 24].

Unexpectedly, (*Z*)‐resveratrol was not detected in our study in the irradiated (*E*)‐resveratrol solution, in contrast to previous photo‐isomerization studies [6, 8, 25, 26]. We believe that the high energy emitted by the artificial solar system used in this study might have led to a most extensive isomerization and subsequent degradation of (*Z*)‐resveratrol. While comparable studies used specific wavelengths for irradiation (e.g., 365 nm^6^, UV‐B^8^), the complex composition of artificial sunlight used in this study may have intensified the degradation of (*E*)‐resveratrol. In this regard, studies suggest that the degradation of (*E*)‐resveratrol occurs more efficiently under combined UV and visible light exposure compared to visible light alone [27]. Notably, in our study, (*Z*)‐resveratrol was found in the irradiated grape cane extract as described in more detail below. Also, triple‐bond resveratrol derivatives (e.g., resveratrone, i.e., 3,4′,5‐trihydroxydiphenylacetylene) were not found in our study in the irradiated (*E*)‐resveratrol sample, although the compound has earlier been reported to be a photodegradation product by Montsko et al. [6].

#### 3.1.2 (*Z*)‐Resveratrol

Similar to irradiated (*E*)‐resveratrol samples, 4‐hydroxybenzaldehyde (Table 1, #B2) was also found in the irradiated (*Z*)‐resveratrol (Table 1, #B0, CCS 154.8 Å^2^, CV 0.01%) sample. Interestingly, herein, further “aldehyde‐like transformation products” [6] were detected as 3,4‐ and 3,5‐dihydroxybenzaldehydes (Table 1, #B1, both [M–H]^−^ at *m/z* 137.0243 equaling C_7_H_5_O_3_^−^, both CCS 118.2 Å^2^). The presence of both 3,4‐ and 3,5‐dihydroxybenzaldehydes was confirmed with authentic standards, being inseparable by both HPLC‐DAD and by TIMS, however, evincing slightly different mass fragmentation pattern (cf. Supporting Information). As revealed by CID experiments of authentic standards, CID of 3,4‐dihydroxybenzaldehyde yielded a distinctive, even‐numbered fragment ion at *m/z* 108.0215 (C_6_H_4_O_2_^•–^, calc. *m/z* 108.0217), possibly generated by radical loss of CHO• from the aldehyde group (see mass fragmentation of 4‐hydroxybenzaldehyde discussed above). By contrast, CID of 3,5‐dihydroxybenzaldehyde resulted in a characteristic fragment ion at *m/z* 93.0365 (C_5_HO_2_^−^, calc. *m/z* 92.9971 or C_6_H_5_O^−^, calc. *m/z* 93.0346), possibly attributed to elimination of C_2_H_4_O (calc. 44.0262 amu) as previously proposed in literature [18] or decarboxylation (loss of CO_2_, calc. *m/z* 43.9898 amu) after rearrangement. As both fragment ions were detected in the HR‐MS/MS spectrum of compound B1 (Table 1), we concluded that both 3,4‐ and 3,5‐dihydroxybenzaldehyde should have been present. Moreover, the irradiated (*Z*)‐resveratrol sample also featured its hydroxylated derivative, i.e., (*E*)‐piceatannol (Table 1, #B3, [M–H]^−^ at *m/z* 243.0665, CCS 159.6 Å^2^) as identified via a reference compound and mass fragmentations discussed below and being in accordance with literature describing the production of (*E*)‐piceatannol [7].

Interestingly, another compound with the exact mass of (*E*)‐piceatannol (Table 1, #B3) was detected, possibly representing (*Z*)‐piceatannol (Table 1, #B6, CCS 156.9 Å^2^). In this regard, the additional hydroxyl moiety (–OH) of *E/Z*‐piceatannol compared to *E/Z*‐resveratrol might explain the observed presence of the dihydroxybenzaldehydes in the irradiated (*Z*)‐resveratrol sample. When irradiating (*E*)‐resveratrol, only the monohydroxylated 4‐hydroxybenzaldehyde was found (see above). The same explanation might be applicable to the occurrence of trihydroxyphenanthrenediones (Table 1, #B4 and B5, [M–H]^−^ at *m/z* 255.0301 to 255.0303, both CCS 147.1 Å^2^, but different retention times) when irradiating the (*Z*)‐isomer instead of the emergence of dihydroxyphenanthrenedione when irradiating (*E*)‐resveratrol, likely stemming from the cyclization of *E/Z*‐piceatannol instead of *E/Z*‐resveratrol as described above.

#### 3.1.3 (*E*)‐Piceatannol

(*E*)‐piceatannol (Table 1 #C0, [M–H]^−^ with C_14_H_11_O_4_^−^ at *m/z* 243.0661, CCS 159.5 Å^2^, CV 0.05%) followed the mass fragmentation pattern of monomeric stilbenoids described above with one and two C_2_H_2_O eliminations, respectively, followed by loss of CO in addition to generation of deprotonated ketene/ethynol. Moreover, a distinctive product ion at *m/z* 225.0565 ([M–H–H_2_O]^−^) was detected that may be indicative of the 3′,4′‐dihydroxylated piceatannol (not being generated during CID of the 4′‐hydroxylated resveratrol).

By analogy to sunlight‐exposed resveratrol samples, the photo‐irradiation of (*E*)‐piceatannol revealed four different photodegradation pathways. Firstly, (*Z*)‐piceatannol (Table 1, #C7, [M–H]^−^ at *m/z* 243.0667, CCS 156.9 Å^2^) was tentatively identified, likely formed through direct isomerization. This identification was supported by matching *m/z* values and HR‐MS/MS fragmentation patterns of the suggested (*Z*)‐isomer with those of the (*E*)‐isomer. In addition, the difference in CCS values between the (*Z*)‐ and (*E*)‐isomer of piceatannol (2.7 Å^2^) aligned with the previously observed CCS shift (2.8 Å^2^) accompanying the *E*/*Z*‐isomerization of resveratrol. We emphasize that signal peaks of (*E*/*Z*)‐isomers of monomeric stilbenoids yielded significantly different CCS values without being baseline‐separated in the mobilogram (Figure S2). Shape and location of the peak maxima were highly reproducible with low coefficients of variation (CV 0.01%–0.75%), confirming that the observed CCS differences provide a robust criterion for differentiation of geometrical isomers, even though the peak bases partly overlap in the mobilogram. These findings clearly highlight that mobilograms represent something fundamentally different from chromatograms, where the horizontal axis is usually a retention time axis. In case of mobilograms, the horizontal axis bears the mobility (1/K_0_, Figure S2).

Secondly, three compounds with deprotonated molecules [M–H]^−^ at *m/z* 485.1239 to 485.1246 (Table 1, #C8/11/13, CCS 207.6, 211.1, and 211.0 Å^2^, resp., all C_28_H_21_O_8_^−^) were attributed to a dimerization of (*E*)‐piceatannol. These putative dimers likely further underwent two different transformations: (i) an oxidation, producing oxidized dimers (possibly phenanthrene‐like compounds as described below for (*E*)‐ɛ‐viniferin degradation) with [M–H]^−^ at *m/z* 483.1085 and 483.1087 (Table 1 #C12/14, CCS 228.2 and 223.8 Å^2^, resp., C_28_H_19_O_8_^−^) and (ii) a cleavage at the double bond, generating 3,4‐dihydroxybenzaldehyde (Table 1, #C2, [M–H]^−^ at *m/z* 137.0245, confirmed using an authentic standard) and its putative counterpart herein referred to as α‐aldehyde (Table 1, #C9, [M–H]^−^ with C_21_H_15_O_7_^−^ at *m/z* 379.0827). These cleavage transformations mirrored those described for (*E*)‐resveratrol and are elaborated below in the section on (*E*)‐ε‐viniferin.

Thirdly, tentative cyclization processes resulted in phenanthrene‐like compounds. Rodriguez‐Cabo et al. [7] have also observed similar products arising from the cyclization of *E*/*Z*‐resveratrol, i.e., tetrahydroxyphenanthrene (Table 1, #C4) and trihydroxyphenanthrenedione (#C5) with [M–H]^−^ at calc. *m/z* 241.0506 and 255.0299, respectively. Both exhibited ion species [M–H–28]^−^ and [M–H–28–44]^−^, representing consecutive losses of CO and CO_2_. The fragmentation pattern for tetrahydroxyphenanthrene (#C4, Table 1) as tentatively observed in our study was in accordance with that of Rodriguez‐Cabo et al. CID of the deprotonated molecules [M–H]^−^ at *m/z* 255.0304 (C_14_H_7_O_5_^−^) of compound #C5 exhibited product ions at *m/z* 227.0362 ([M–H–28]^−^) and 183.0557 ([M–H–28–44]^−^) from the putative eliminations of CO and CO_2_. All measured *m/z* were 16 Da higher than those of resveratrol due to the presence of an additional hydroxy group in piceatannol. Therefore, compound #C5 was tentatively identified as trihydroxyphenanthrenedione. Compound #C6 carrying an additional oxygen ([M–H]^−^ at *m/z* 271.0248 equaling C_14_H_7_O_6_^−^) displayed a similar fragmentation pattern with product ions at *m/z* 243.0303 ([M–H–28]^−^) and 227.0372 ([M–H–44]^−^) in the HR‐MS/MS spectrum, which has been previously reported for the resveratrol transformation product tetrahydroxyphenenthrenedione (transformation product TP36 in Rodriguez‐Cabo et al. [7]).

Finally, the presence of 3,4‐dihydroxybenzaldehyde (Table 1, #C2, [M–H]^−^ at *m/z* 137.0245) was confirmed by an authentic external standard. An unspecified dihydroxybenzoic acid (Table 1, #C1, [M–H]^−^ at *m/z* 153.0193 equaling C_7_H_5_O_4_^−^) was tentatively assigned on the basis of its short retention time matching that of authentic reference standards of 3,5‐ and 3,4‐dihydroxybenzoic acids (both *t*_R_ = 2.5 min). Notably, the reference standards (cf. Supporting Information) were not resolved applying the HPLC method detailed above and displayed similar CCS values of 124.0 and 121.3, respectively. HR‐MS/MS spectra displayed the common product ions from putative decarboxylation of the carboxy group at calc. *m/z* 109.0295 ([M–H–CO_2_]^−^, C_6_H_5_O_2_^−^), equaling deprotonated dihydroxybenzene (most likely resorcinol and catechol, respectively). Further common product ions were detected at calc. *m/z* 65.0033 (C_4_HO^−^) and 41.0034 (C_2_HO^−^, equaling deprotonated ketene/ethynol). However, in the HR‐MS/MS experiment, 3,5‐dihydroxybenzoic acid displayed distinctive fragment ions at *m/z* 67.0188 (C_4_H_3_O^−^ calc. *m/z* 67.0189) whereas CID of 3,4‐dihydroxybenzoic acid generated indicative fragment ions at *m/z* 108.0214 (C_6_H_4_O_2_^•–^ calc. *m/z* 108.0217, see also CID of 3,4‐dihydroxybenzaldehyde) and 91.0187 (C_6_H_3_O^−^ calc. *m/z* 91.0189). These three fragment ions have been previously proposed to differentiate resorcinol and catechol [28]. As all of the aforementioned product ions were detected in the HR‐MS/MS spectrum of #C1, we may conclude that both 3,5‐ and 3,4‐dihydroxybenzoic acid were present. This indicated a photo‐induced direct cleavage of (*E*)‐piceatannol followed by ring‐opening into the respective acid as described in detail for resveratrol. These compounds carried one additional oxygen atom in their structure than their counterparts derived from resveratrol and its di−/tetramers assessed (i.e., 4‐hydroxybenzaldehyde), and may originate from the cleavage of piceatannol but also its dimers mentioned above, making the pathway assignment challenging.

#### 3.1.4 (*E*)‐Piceid (Resveratrol 3‐*O*‐β‐D‐glucopyranoside)

CID of the glycosylated (*E*)‐resveratrol derivative (*E*)‐piceid (Table 1, #D0, [M–H]^−^ at *m/z* 389.1242, C_20_H_21_O_8_^−^) resulted in the deprotonated aglycone ([M–H–162]^−^) after elimination of the glucosyl moiety (C_6_H_10_O_5_, calc. 162.0528 amu) in addition to fragment ions also found in the HR‐MS/MS spectrum of resveratrol. After irradiation, we tentatively identified a dihydroxyphenanthrenedione (Table 1, #D10, [M–H]^−^ at *m/z* 239.0352) and a putative hydroxyphenylethinyl butenedioic acid anhydride (Table 1, #D11, [M–H]^−^ at *m/z* 213.0195) following the above‐described identification strategy. Their genesis may be explained in agreement with those of the degradation products of (*E*)‐resveratrol, here additionally including a hydrolytic elimination of (*E*)‐piceid's sugar moiety.

Likewise, the detection of 4‐hydroxybenzaldehyde (Table 1, #D6) agreed with the findings made with the irradiated (*E*)‐resveratrol sample. Additionally, we detected putative glucosides of hydroxybenzoic acids and/or dihydroxybenzaldehydes (Table 1 #D5/7, [M–H]^−^ at *m/z* 299.0775 to 299.0776, CCS 171.7 and 169.2 Å^2^, resp.), as identified according to a characteristic loss of a hexosyl moiety [M–H–162]^−^ resulting in the aglycon at *m/z* 137.0246 and 137.0243, respectively (both C_7_H_5_O_3_^−^, calc. *m/z* 137.0244). Hereby, the fragment ions at *m/z* 93.0346 and 93.0350, respectively, rather support the assignment as glucosylated dihydroxybenzaldehydes as detailed for the non‐glucosylated analogues described above. However, the assignment of #D5/7 being properly resolved by chromatography remains somehow obscure and we cannot conclusively distinguish between dihydroxybenzaldehyde‐ and hydroxybenzoic acid‐*O*‐glucosides or assign them to compound #D5 or compound #D7, respectively. Nevertheless, these compounds may represent glycosylated derivatives of the “aldehyde‐like transformation products” mentioned earlier by Rodriguez‐Cabo et al. [7]. This also applies to the dihydroxybenzoic acid‐*O*‐glucoside that displayed two abundant mobilities each with identical mass fragmentations (Table 1, #D3/4, both [M–H]^−^ at *m/z* 315.0726, CCS 167.6 and 175.2 Å^2^, resp.). CID generated base peak fragment ions at *m/z* 153.0197 and 153.0183 ([M–H–162]^−^), equaling a deprotonated dihydroxybenzoic acid (C_7_H_5_O_4_^−^, calc. *m/z* 153.0193). This assignment was substantiated by the product ions at *m/z* 109.0296 (C_6_H_5_O_2_^−^, calc. *m/z* 109.0295) that may resemble deprotonated 1,3‐dihydroxybenzol (resorcinol) resulting from decarboxylation of the carboxy group and at *m/z* 67.0194 to 67.0198 (C_4_H_3_O^−^, calc. *m/z* 67.0189) that both were also detected in the HR‐MS/MS spectrum of an authentic 3,5‐dihydroxybenzoic acid standard (cf. Supporting Information).

To our knowledge, Gonzales et al. [29] have been to date the only ones to report a measured mobility from drift tube IMS for a stilbenoid, specifically resveratrol‐3‐*O*‐glucoside (piceid), with a CCS of 197.7 Å^2^. The discrepancy between this value and our measured CCS (182.1 Å^2^) might be attributed to different types of IMS instrumentation used. Drift tube IMS used by Gonzales et al. [29] drives ions through a stationary gas, while TIMS holds the ions stationary in a moving column of gas [30], possibly contributing to differences described above. Further systematic research including the direct juxtaposition of these instrumentations warrants the precise elucidation of such discrepancies.

### 3.2 Degradation of Dimeric Stilbenoids

#### 3.2.1 (*E*)‐Ampelopsin A

The dimeric stilbenoid (*E*)‐ampelopsin A (Table 1, #E0, [M–H]^−^ at *m/z* 469.1296) displayed characteristic HR‐MS/MS fragment ions at *m/z* 451.1136 ([M–H–H_2_O]^−^) and 363.0853 ([M–H–106]^−^) from the neutral losses of water and a C_7_H_6_O (calc. 106.0418 amu) moiety, respectively, as previously reported to occur during CID of the dimeric stilbenoid (*E*)‐ɛ‐viniferin [16]. Upon irradiation, #E0 showed the lowest number of detectable photodegradation products of all tested stilbenoids. Specifically, aside from the parent compound #E4, the irradiated sample yielded a dihydroxy‐ and 4‐hydroxybenzoic acid (Table 1, #E1/2) along with 4‐hydroxybenzaldehyde (Table 1, #E3). Notably, 4‐hydroxybenzoic acid was exclusively found after illumination of di‐ and tetrameric stilbenoids. CID of the deprotonated molecules [M–H]^−^ of the authentic standard of 4‐hydroxybenzoic acid at *m/z* 137.0244 generated odd‐numbered fragment ions at *m/z* 109.0303 ([M–H–CO]^−^), 93.0322 ([M–H–CO_2_]^−^), 91.0192, and 41.0041 as well as even‐numbered ions at *m/z* 108.0255 and 92.0263 (cf. Supporting Information). These compounds likely originated from hydroxylation and cleavage of a monomeric stilbenoid, potentially resulting from direct splitting of the dimer, as discussed above. The limited number of photodegradation products observed for (*E*)‐ampelopsin A may be linked to its planar structure [31], which might have contributed to increased stability, potentially reducing susceptibility to photodegradation. Thus, (*E*)‐ampelopsin A's structural features may play a role in its relatively low degradation rate under irradiation, warranting further investigation into how structural factors influence stilbenoid stability.

#### 3.2.2 (*E*)‐ɛ‐Viniferin

Photodegradation of (*E*)‐ɛ‐viniferin (Table 1, #F0, [M–H]^−^ at *m/z* 453.1344, indicative fragment ion [M–H–106]^−^ at *m/z* 347.0938 as discussed above) under artificial sunlight indicated three distinct degradation pathways. The first pathway involved the cleavage of the dimer into monomeric units, i.e., *E*/*Z*‐resveratrol, which subsequently underwent further photodegradation. Evidence for this includes the detection of tentative phenanthrene‐like compounds such as dihydroxyphenanthrenedione (Table 1, #F7) and trihydroxyphenanthrenediones (Table 1, #F8/9). The emergence of these compounds, also identified as photodegradation products of (*E*)‐ and (*Z*)‐resveratrol, suggested that the monomers produced from the cleavage were degraded via known mechanisms. Interestingly, the putative trihydroxyphenanthrenediones observed at *m/z* 255.0300 showed retention times (*t*_R_) and fragmentation patterns differing from analogous products in irradiated monomers, hinting at the formation of isomers with varying hydroxy moiety positions as suggested by Rodriguez‐Cabo et al. [7]. Beyond this, the observation of two different mobilities (148.9 and 153.5 Å^2^) displayed for the signals monitored at *m/z* 255.0300 at *t*_R_ 7.3 min raised the hypothesis that two co‐eluting isomers of trihydroxyphenanthrenediones had been present. Furthermore, a putative pentahydroxyphenanthrenedione (Table 1, #F5, *m/z* 257.0460) was detected with fragmentation patterns consistent with this class of compounds. The detection of cleavage products like hydroxyphenylethinyl butenedioic acid anhydride (Table 1, #F10) and aldehydes such as dihydroxybenzaldehyde (#F1), 4‐hydroxybenzaldehyde (#F3), and 4‐hydroxybenzoic acid (#F2) further supported this pathway, as these products aligned with known degradation patterns of monomeric stilbenoids.

The second suggested pathway involved direct cleavage of the dimer at the ethylene bridge, forming unique products that are not linked to monomeric precursors (cf., Figure 1). A significant compound possibly arising from this mechanism was an “α‐aldehyde” (Table 1, #F6, *m/z* 363.0874), proposed to result from the cleavage at the double bond. Its corresponding “α‐acid” (Table 1, #F4, *m/z* 379.0825) exhibited the typical fragmentation pattern [M–H–44]^−^ at *m/z* 335.0929 and [M–H–106]^−^ at *m/z* 273.0414, representing the loss of a CO_2_ and C_7_H_6_O moiety, respectively. The naming of these compounds as α‐aldehyde and α'‐aldehyde is based on the relative partial charges of the two sp^2^‐hybridized carbons at the double bond, with the carbon bearing the more electronegative substituents (or influenced by adjacent functional groups) designated as α, and that with less electronegative substituents as α'. In the case of (*E*)‐ɛ‐viniferin, the cleavage yielded 4‐hydroxybenzaldehyde (Table 1, #F3), herein representing an α'‐aldehyde, and a counterpart α‐aldehyde at *m/z* 363.0874 (Table 1, #F6). Interestingly, the direct cleavage pathway mirrors that observed in the photodegradation of monomeric resveratrol, reinforcing the shared degradation mechanisms between monomeric and dimeric stilbenoids.

The third pathway indicates an oxidative photocyclization resulting in phenanthrene derivatives of ɛ‐viniferin as already reported by Pébarthé‐Courrouilh et al. [17]. The further oxidation of ɛ‐viniferin phenanthrene (Table 2, #21) generated the tentatively assigned ɛ‐viniferin dihydroxyphenanthrenedione in the irradiated stock solution (Table 1, #F11). The latter compound with [M–H]^−^ at *m/z* 481.0930 displayed fragment ions at *m/z* 453.0985 ([M–H–28]^−^), 437.1057 ([M–H–44]^−^), 409.1103 ([M–H–28–44]^−^), and 331.0590 ([M–H–44–106]^−^) resembling the fragmentation pattern of trihydroxyphenanthrenediones (Table 1, #B4 and #B5) with losses of CO and CO_2_ in addition to the elimination of a C_7_H_6_O moiety as described above.

**TABLE 2:** HPLC‐DAD‐ESI(−)‐TIMS‐QTOF‐HR‐MS/MS of phenolic compounds with due regard to stilbenoids in the grape cane extract before (a) and after (b) photo‐irradiation.

#	Compound	*t*_R_ (min)	λ_max_ (nm)	Exp. mass [M–H]^−^ (*m/z*)	Calc. mass [M–H]^−^ (*m/z*)	Error (ppm)	Sum formula	ESI(−)‐HR‐MS/MS (*m/z* (% base peak intensity))	^TIMS^CCS_N2_ (Å^2^)	Mobility 1/K_0_ (Vs/cm^2^)
1	Procyanidin dimer^a^, ^b^	2.7	278	577.1346	577.1351	1.0	C_30_H_25_O_12_^−^	425.0866 (50), 407.0781 (27), 289.0724 (100), 125.0244 (40)	222.0	1.082
2	*p*‐coumaric acid hexose^a^, ^b^	2.8	nd	325.0927	325.0929	0.5	C_15_H_17_O_8_^−^	163.0404 (82), 119.0501 (100)	163.8	0.785
3	3,4‐ & 3,5‐dihydroxybenz‐aldehyde^§^ ^,^ ^b^	3.0	260	137.0244	137.0244	−0.1	C_7_H_5_O_3_^−^	108.0208 (12), 93.0350 (10), 41.0033 (12)	118.4	0.539
4	4‐hydroxybenzaldehyde^§^ ^,^ ^b^	3.9	284	121.0298	121.0295	−2.4	C_7_H_5_O_2_^−^	92.0269 (32), 41.0439 (5)	116.6	0.525
5	Quercetin‐3‐*O*‐hexuronide^a^, ^b^	4.0	nd	477.0669	477.0675	1.1	C_21_H_17_O_13_^−^	301.0355 (100), 178.9985 (8), 151.0032 (24), 121.0292 (5)	203.5	0.988
6	(*E*)‐piceid^§^ ^,^ ^a^	4.1	nd	389.1242	389.1242	0.0	C_20_H_21_O_8_^−^	227.0721 (100), 185.0610 (5), 143.0506 (10)	182.2	0.879
7	(*E*)‐ampelopsin A^§^ ^,^ ^a^, ^b^	4.5	281	469.1291	469.1293	0.3	C_28_H_21_O_7_^−^	451.1255 (50), 375.0880 (50), 363.0910 (71)	203.0	0.985
	‐ Formic acid adduct			515.1347	515.1348	−0.1	C_29_H_23_O_9_^−^	469.1279 (100), 451.1200 (29)	213.4	1.038
8	(*E*)‐piceatannol^§^ ^,^ ^a^	4.7	nd	243.0664	243.0663	0.3	C_14_H_11_O_4_^−^	225.0551 (4), 185.0610 (5), 143.0506 (10)	159.5	0.754
9	Stilbenoid dimer^a^, ^b^	5.2	280	453.1342	453.1344	0.2	C_28_H_21_O_6_^−^	359.0947 (100), 93.0343 (25)	nd	nd
	‐ Formic acid adduct			499.1401	499.1398	−0.4	C_29_H_23_O_8_^−^	453.1332 (100), 359.0920 (61), 93.0348 (17)	218.9	1.061
10	(*E*)‐resveratrol^§^ ^,^ ^a^, ^b^	5.8	305, 320	227.0715	227.0714	−0.5	C_14_H_11_O_3_^−^	185.0603 (16), 143.0502 (14), 115.0557 (8), 41.0042 (8)	157.7	0.743
11	Stilbenoid (*E*)‐dimer^a^	6.3	280	453.1342	453.1344	0.4	C_28_H_21_O_6_^−^	359.0946 (52), 93.0343 (13)	nd	nd
	‐ Formic acid adduct			499.1393	499.1398	1.0	C_29_H_23_O_8_^−^	453.1342 (100), 359.0920 (55), 93.0348 (10)	223.6	1.087
12	Stilbenoid (*Z*)‐dimer^b^	6.3	279	453.1342	453.1344	0.4	C_28_H_21_O_6_^−^	359.0982 (100), 105.0330 (100), 93.0328 (97)	nd	nd
	‐ Formic acid adduct			499.1393	499.1398	1.0	C_29_H_23_O_8_^−^	453.1342 (100)	213.3	1.034
13	Hopeaphenol^§^ ^,^ ^a^, ^b^	6.5	281	905.2595	905.2604	0.9	C_56_H_41_O_12_^−^	—	286.0	1.407
14	Isohopeaphenol^a^, ^b^	6.8	281	905.2595	905.2604	0.9	C_56_H_41_O_12_^−^	—	286.9	1.411
15	(*Z*)‐resveratrol^§^ ^,^ ^b^	7.0	284	227.0714	227.0714	−0.1	C_14_H_11_O_3_^−^	185.0613 (20), 143.0504 (20), 115.0553 (7), 41.0042 (8)	155.4	0.732
16	Trihydroxyphenanthrene	7.1	260	225.0554	225.0557	1.4	C_14_H_9_O_3_^−^	183.0452 (4), 181.0660 (10), 157.0658 (2), 41.0043 (3)	146.6	0.690
17	(*Z*)‐ɛ‐viniferin^b^	7.3	282	453.1342	453.1344	0.4	C_28_H_21_O_6_^−^	347.0937 (3), 253.0527 (2), 41.0040 (2)	206.4	1.000
	‐ Formic acid adduct			499.1403	499.1398	−0.9	C_29_H_23_O_8_^−^	453.1348 (100)	218.4	1.061
18	(*E*)‐ɛ‐viniferin^§^ ^,^ ^a^, ^b^	7.5	322	453.1342	453.1344	0.4	C_28_H_21_O_6_^−^	347.0937 (6)	206.3	1.000
	‐Formic acid adduct			499.1399	499.1398	−0.2	C_29_H_23_O_8_^−^	453.1340 (100)	218.9	1.064
19	Stilbenoid trimer^a^, ^b^	7.7	284, 320	679.1973	679.1974	0.1	C_42_H_31_O_9_^−^	nd	nd	nd
	‐ Formic acid adduct			725.2022	725.2028	0.9	C_43_H_33_O_11_^−^	679.1964 (100)	250.9	1.228
20	(*E*)‐r2‐viniferin^§^ ^,^ ^a^	7.9	282, 324	905.2599	905.2604	0.5	C_56_H_41_O_12_^−^	nd	nd	nd
21	ɛ‐viniferin phenanthrene	8.0	271	451.1186	451.1187	0.3	C_28_H_19_O_6_^−^	225.0568 (2), 224.0475 (3), 93.0343 (1)	217.1^c^	1.052^c^
22	Stilbenoid dimer^a^	8.1	320	453.1333	453.1344	2.3	C_28_H_19_O_6_^−^	nd	nd	nd
	‐ Formic acid adduct			499.1389	499.1398	−1.8	C_29_H_23_O_8_^−^	453.1331 (100)	215.5	1.047
23	(*E*)‐r‐viniferin^§^ ^,^ ^a^	9.1	323	905.2603	905.2604	−0.4	C_56_H_41_O_12_^−^	—	nd	nd

Abbreviations: CCS, collision cross section; nd, not determined; *t*_R_, retention time; λ_max_, UV/Vis absorption maxima.

^a^ Found in the grape cane extract before irradiation.

^b^ Found in the grape cane extract after irradiation.

^c^ CCS and mobility tentatively assigned.

^§^ Confirmed by an external standard.

Notably, dimeric stilbenoids, including (*Z*)‐ɛ‐viniferin, were not detected in our irradiated samples, diverging from earlier findings by Francioso et al. [32] and Kosovic et al. [25], who reported the formation of (*Z*)‐ɛ‐viniferin through photoisomerization or dimerization. This discrepancy may stem from the higher energy output of our artificial sunlight source, which achieved complete decomposition of stilbenoids within 2 h, compared to the extended degradation over 20 days in Kosovic et al.'s study. Additionally, in their experiments, the production of (*Z*)‐ɛ‐viniferin may have predominantly arisen from (*E*)‐resveratrol dimerization rather than direct isomerization of (*E*)‐ɛ‐viniferin. This possibility aligns with our observations of stilbenoid dimer formation from resveratrol. These findings underline the complexity of (*E*)‐ɛ‐viniferin degradation and highlight the need for further systematic studies to unravel the detailed mechanisms involved.

### 3.3 Degradation of Tetrameric Stilbenoids

#### 3.3.1 Hopeaphenol

Photodegradation products found in the irradiated hopeaphenol (Table 1, #G0, [M–H]^−^ at *m/z* 905.2610) sample were dihydroxy‐ and 4‐hydroxybenzoic acid (Table 1, #G1/2) as well as 4‐hydroxybenzaldehyde (Table 1, #G3) along with the parent compound itself. Additionally, (*E*)‐ampelopsin A (Table 1, #G4, CCS 203.0 Å^2^) was detected as identified by using an authentic standard, being present in the irradiated and, in lower and generally small amounts, also in the untreated sample, suggesting a contamination or insufficient purification of the hopeaphenol standard. It is conceivable that the origin of benzaldehyde or benzoic acid related degradation products found in this sample might have been attributable to the photodegradation of both, hopeaphenol or the observed traces of (*E*)‐ampelopsin A, particularly as they had been observed in the respective irradiation experiment of (*E*)‐ampelopsin A before (see above).

The stilbenoid family specific formula ([M–H]^−^ of C_28_H_19_O_6_^−^, i.e., differing by one H_2_O from, e.g., ampelopsin A) led us to the proposition that “unknown compound 4” (#G6) might represent a structure similar to that of, e.g., viniferifuran (i.e., amurensin H) [33]. To our knowledge, this compound has not yet been reported as a photodegradation product of hopeaphenol, but has instead been associated with being natively present in stems of *Vitis amurensis* rupr., roots of *Vitis thunbergii* siebold & zucc., and stems of *V. vinifera* [34, 35, 36]. Therefore, our assumption warrants further verification through additional identification steps, including the use of authentic standards, which are currently unavailable for viniferifuran.

#### 3.3.2 (*E*)‐r2‐Viniferin

The irradiation of (*E*)‐r2‐viniferin (i.e., vitisin A) showed a degradation pattern similar to those observed for mono‐ and dimeric stilbenoids. First, the presence of dihydroxy‐ and 4‐hydroxybenzaldehyde along with their respective acids (Table 1, #H1–4) affirmed the photodegradation “cleavage” pathway into “aldehyde‐like transformation products” seen in all tested samples and described above.

In contrast to observations in irradiated mono‐ and dimeric stilbenoid samples, artificial sunlight exposure of (*E*)‐r2‐viniferin led to the formation of a novel compound (#H9) with the same [M–H]^−^ at *m/z* 905.2602 (C_56_H_41_O_12_^−^) as the parent compound (Table 1, #H0/H10). This compound was tentatively identified as (*Z*)‐r2‐viniferin. The presumed isomers were distinguished by differences in retention time, λ_max_, and collisional cross‐section (CCS) values. Specifically, the (*E*)‐isomer exhibited a λ_max_ of 323 nm and a CCS of 285.1 Å^2^ (Table 1, #H0), whereas the tentative (*Z*)‐isomer showed a slightly higher CCS of 289.3 Å^2^ (Table 1, #H9), alongside a distinct shorter retention time and a lower λ_max_ of 282 nm as also described for other (*Z*)‐isomers. Furthermore, an “α‐aldehyde” (Table 1 #H7, [M–H]^−^ at *m/z* 363.0878, C_21_H_15_O_6_^−^), as already described in the irradiated (*E*)‐ɛ‐viniferin sample (cf. #F7), might also indicate a direct cleavage of the tetramer at the double‐bond (cf., Figure 1). This might be underlined by the occurrence of this cleavage's counterpart, i.e., an α'‐aldehyde (Table 1, #H8, [M–H]^−^ at *m/z* 573.1558, C_35_H_25_O_8_^−^) as well as the respective α‐ and α'‐acids (Table 1, #H5/H6, [M–H]^−^ at *m/z* 379.0826 and 589.1505 equaling C_21_H_15_O_7_^−^ and C_35_H_25_O_9_^−^, respectively), identified via their shorter *t*_R_ compared to those of the corresponding aldehydes and the typical decarboxylation fragmentation pattern evincing a loss of 44 Da as described in the (*E*)‐ɛ‐viniferin section.

More intriguingly, an additional compound detected as [M–H]^−^ at *m/z* 573.1550 (Table 1, #H11, CCS 234.8 Å^2^) indicated the formation of another “α'‐aldehyde‐like compound” with the same formula as #H8, featuring a different retention time, mobility, and fragmentation pattern than the one described above. Despite its unspecific fragmentation pattern, the lack of adequate literature and the absence of an authentic standard, one could speculate that one of the aldehydes #H8 or #H11 may be viniferal, featuring a specific mass in the stilbenoid class as reported earlier [37]. The occurrence of such α‐ and α'‐aldehydes could reinforce our hypothesis that the “cleavage pathway” previously described for resveratrol, and further demonstrated in this study, also applies to stilbenoid dimers and tetramers (cf., Figure 1).

#### 3.3.3 (*E*)‐r‐Viniferin

Two of the above‐mentioned aldehydes, i.e., 3,5‐dihydroxy‐ and 4‐hydroxybenzaldehyde, along with their corresponding acids (Table 1, #I1–4) were detected after irradiation of (*E*)‐r‐viniferin (Table 1, #I0). As a limitation of our study, we emphasize that our isolated (*E*)‐r‐viniferin sample had contained an impurity of (*E*)‐resveratrol (Table 1, #I7), present also in the untreated solution. Consequently, the aldehydes and acids mentioned above, as well as the presence of (*Z*)‐resveratrol (Table 1, #I8) might not be directly related to (*E*)‐r‐viniferin degradation, as they could have emerged from the degradation and isomerization of (*E*)‐resveratrol. Nevertheless, the emergence of compounds #I5, #I6, #I9, and #I10, i.e., the abovementioned α‐ and α'‐aldehydes and their respective acids, is consistent with the findings from the irradiated (*E*)‐r2‐viniferin sample, which had not contained the aforementioned resveratrol impurity. The r‐viniferin photodegradation compound #I9, the suggested α'‐acid produced from irradiating (*E*)‐r‐viniferin was highly similar to the suggested α'‐acid of irradiated (*E*)‐r2‐viniferin (#H6) according to its mass spectra, but was different in terms of retention time (7.2 vs. 5.4 min for α'‐acids deriving from r‐ and r2‐viniferin, resp.) and CCS (237.3 vs. 236.5 Å^2^). Therefore, we suppose that the two degradation products are not identical and further study is needed for clarification.

### 3.4 Degradation of the Grape Cane Extract Constituents

**FIGURE 2:**
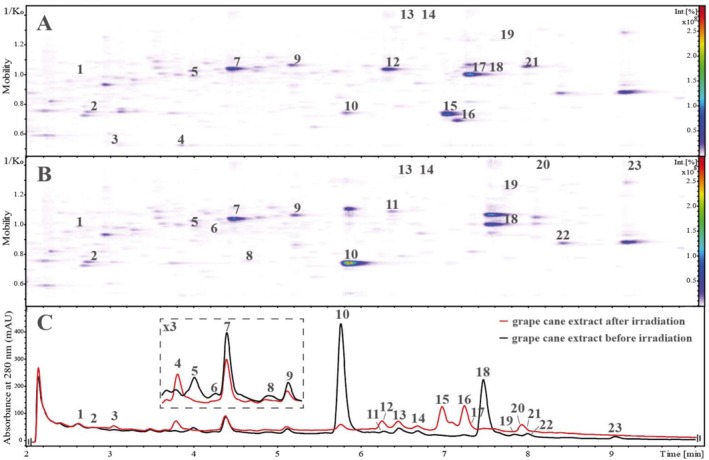
HPLC‐DAD‐ESI(−)‐TIMS‐HR‐MS analyses of phenolic compounds of the grape cane extract: Heatmap after (A) and before (B) photo‐irradiation as well as the corresponding chromatograms at 280 nm (C).

All investigated parent stilbenoids were identified in the grape cane extract. Table 2 lists stilbenoidic and further phenolic compounds present in the extract before (a) and after (b) photo‐irradiation. Figure 2 displays the HPLC‐DAD chromatogram (C) of the grape cane extract along with the corresponding ion mobility heatmaps, where the retention time (*t*_R_) was plotted against the inverse ion mobility (1/K_0_), and the color intensity represents the ion mobilogram's intensity before (B) and after (A) irradiation.

In the untreated grape cane extract, all nine stilbenoids studied in this work were identified by comparison of their *t*_R_ as well as their UV absorption and mass spectral data, including CCS values, with those obtained using authentic reference compounds (Table 2).

Consistent with observations in all standard solutions, photo‐irradiation of the grape cane extract mainly resulted in compounds detectable as [M–H]^−^ at calc. *m/z* 137.0244 and 121.0295 (Table 2, #3 and #4), presumed to be 4‐hydroxybenzaldehyde and 3,4‐/3,5‐dihydroxybenzaldehydes, as described above and verified via reference compounds. Compound #16 with molecular ions [M–H]^−^ at *m/z* 225.0554 (C_14_H_9_O_3_^−^, calc. *m/z* 225.0557, CCS 146.6 Å^2^) displayed fragment ions at *m/z* 183.0452 (C_12_H_7_O_2_^−^, [M–H–42]^−^), 181.0660 (C_13_H_9_O^−^, [M–H–44]^−^), 157.0658 (C_11_H_9_O^−^, [M–H–68]^−^), and 41.0043 (C_2_HO^−^, i.e., deprotonated ketene/ethynol). In addition to ketene elimination described for all constituents carrying resorcinol moieties, CID resulted in eliminations of carbon dioxide (CO_2_, calc. 43.9898 amu) and carbon suboxide (C_3_O_2_, calc. 67.9898 amu). Notably, decarboxylation requires ring‐opening of the tautomeric keto form of the resorcinol moiety followed by generation of a lactone as an intermediate as previously proposed in literature [38, 39]. The uncommon fragment ions generated by the neutral loss of carbon suboxide (O=C=C=C=O) have been previously reported during CID of resveratrol [38], but also flavones [40] in both instances resulting from cleavage of the resorcinol ring. Compound #16 possibly representing a trihydroxyphenanthrene may indicate the “cyclization” pathway to be relevant for stilbenoid degradation in the extract (Figure 1). This assignment is supported by the UV absorption spectrum with a main maximum at 260 nm (together with a shoulder at 315 and low abundant maxima at 343 and 361 nm, not shown in Table 2) resembling that of 2,4,6‐trihydroxyphenanthrene unambiguously identified as a resveratrol degradation product by NMR spectroscopy [32]. Alternatively, #16 may be assigned to 3,4′,5‐trihydroxydiphenylacetylene, resulting from oxidation of the central double bond of resveratrol; however, both constituents can be clearly differentiated by their UV maxima as proposed by Montsko et al. [6]. Notably, compound #16 was not found in the irradiated stilbenoid stock solutions.

Conversely, the related quinones as observed in irradiated standards were not detected after irradiating the grape cane extract. Their apparent absence does not necessarily mean that they had not been formed, because the commonly highly reactive quinones might have reacted with nucleophilic constituents of the extracts. In the irradiated extract, most studied standard stilbenoids were still also detected, except for (*E*)‐piceid as well as r‐ and r2‐viniferin, possibly due to their low quantities preexisting in the untreated extract. Both (*E*)‐resveratrol and (*E*)‐ɛ‐viniferin also evinced only a minimal peak, although being the most abundant stilbenoids in the extract, indicating their susceptibility to irradiation.

At this point, it is important to stress that our efforts in this work concentrated on the qualitative analysis of stilbenoids, not quantifying levels of the respective compounds. Nevertheless, (*Z*)‐resveratrol—not present in any of the irradiated standard solutions mentioned above—was found in the grape cane extract after light exposure. This observation is in accordance with previous literature [12, 32]. As mentioned above, (*Z*)‐resveratrol may be readily photodegraded by high‐energy light and UV radiation to further products described above, thus not being present in irradiated solutions of individual, pure standards. The grape cane extract, however, features a brown matrix that might have provided a certain degree of protection against light degradation. Stabilizing effects of plant extracts on the light‐induced decay of stilbenoids have been suggested previously by Latva‐Mäenpää et al. [41].

Another explanation for the observed (*Z*)‐resveratrol in the grape cane extract may lie in the significantly lower concentration of (*Z*)‐resveratrol in the extract (0.1 mM) [12] compared to the standard solution (1.6 mM), which may have facilitated rapid photodegradation of any (*Z*)‐resveratrol formed into further degradation products as described earlier.

A further putative (*Z*)‐isomer, possibly (*Z*)‐ɛ‐viniferin (Table 2, #17, CCS 206.4 Å^2^), was absent in the irradiated solutions of the respective (*E*)‐isomer but apparently present in the irradiated extract. The compound was tentatively identified by elucidating its sum formula (C_28_H_21_O_6_^−^, exp. *m/z* 453.1342, calc. *m/z* 453.1344) and characteristic fragment ions [M–H–106]^−^ at *m/z* 347.0937 and [M–H–106–94]^−^ at *m/z* 253.0527, representing the sequential loss of C_7_H_6_O and C_6_H_6_O. This fragmentation behavior has been previously reported [42]. In addition, the UV absorption maximum at 282 nm, differing widely from that of its (*E*)‐isomer at 322 nm, supported the presence of the (*Z*)‐isomer. Conceivable production pathways of (*Z*)‐ɛ‐viniferin in the irradiated grape cane extract include (a) a dimerization of (*E*)‐resveratrol, (b) an isomerization of (*E*)‐ɛ‐viniferin, and (c) the gradual degradation of r2‐viniferin. Interestingly, the untreated extract also featured also dimeric stilbenoids such as e.g., (*E*)‐ɛ‐viniferin ([M–H]^−^ with C_28_H_21_O_6_^−^, calc. *m/z* of 453.1344), but with different *t*_R_ and CCS values than those found in the stock solutions (Table 2, #12/22).

Several dimeric stilbenoids were detected in the irradiated sample, including an oxidized species (Table 2, #21) with a sum formula indicating the loss of two hydrogen atoms compared to the other dimers (Table 2, #21, [M–H]^−^ of C_28_H_19_O_6_^−^ at calc. *m/z* 451.1187, CCS 217.1 Å [2]). Compound #21 may possibly represent an ε‐viniferin phenanthrene derivative, unambiguously identified as a phototransformation product of (*E*)‐ε‐viniferin generated via (*Z*)‐ε‐viniferin following oxidative photocyclization [17]. This may be supported by the UV absorption spectrum (main λ_max_ at 271 nm in addition to low‐abundant maxima at 353 and 370 nm, not shown in Table 2), and a spectral shape resembling that of a trihydroxyphenanthrene (#17). Based on consistent λ_max_ and HR‐MS/MS data, some of the dimers might tentatively be attributed to known grape cane constituents such as pallidol and ω‐viniferin. However, due to the absence of authentic reference standards, a definitive structural assignment is currently not possible [34, 43]. Furthermore, non‐stilbenoidic compounds were detected in both the untreated and the irradiated grape cane extract, i.e., a procyanidin dimer (Table 2, #1), a *p*‐coumaric acid hexose (i.e., a *p*‐coumaric acid‐4‐*O*‐hexoside or a *p*‐coumaroylhexose, #2), and a quercetin‐3‐*O*‐hexuronide (#5), possibly a glucuronide as previously reported [34], but not further investigated beyond reporting their CCS values and HR‐MS/MS data herein.

### 3.5 Considerations on Ion Mobility Measurements

**FIGURE 3:**
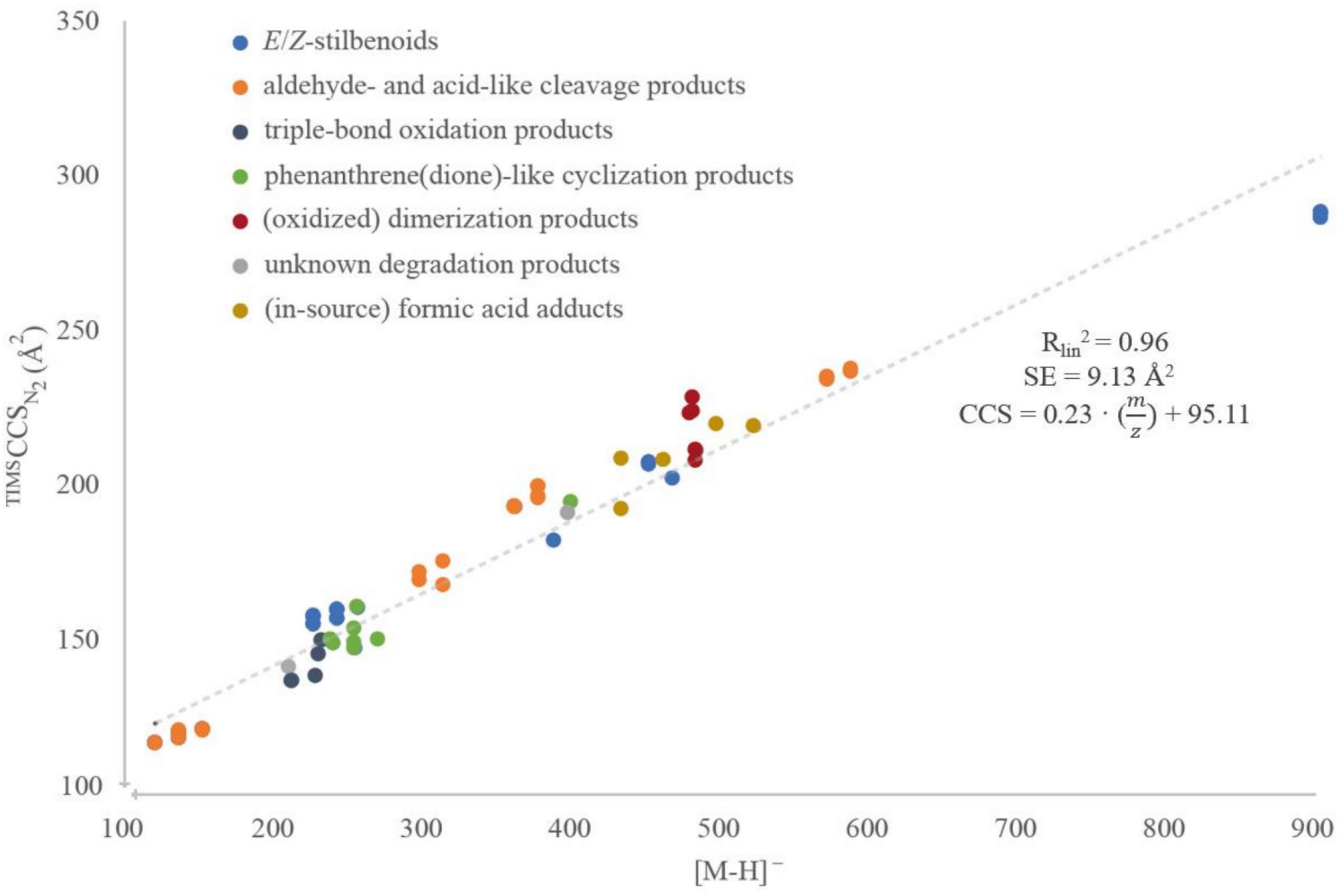
Relation between measured *m/z* of the molecular ions [M–H]^−^ and collision cross section (CCS) values for all stilbenoids and their degradation products from Table 1 labeled according to the proposed degradation pathway with the coefficient of determination (*R* ^2^) and standard error of the estimate (SE).

CCS values of measured stilbenoids and their photodegradation products (Table 1, [M–H]^−^ at *m/z* 121–905) identified in this study were found in the range of 116.5 to 286.4 Å^2^. The precision of the TIMS measurement was assessed by triplicate injections of the authentic standards, yielding coefficients of variation between 0.01% and 0.75%. These values are reported in Tables 1 and S1. The expected correlation between the mass of the molecular ions [M–H]^−^ and their CCS values is displayed in Figure 3, confirming that ion mobility of an analyte is clearly correlated with its mass (*R*_lin_^2^ = 0.96). The standard error of estimate (SE = 9.13 Å^2^) reflects the average deviation of experimental CCS values from the regression line, indicating that additional structural factors beyond molecular mass influence ion mobility. Differences in CCS values of isobaric compounds further support this conclusion. While the absolute accuracy of IMS measurements across different platforms and labs is often cited within a 2%–3% range [14, 44], the high intra‐instrumental precision achieved here allows for the reliable resolution of isomeric stilbenoids. However, considering the calibration accuracy and observed CCS variability, CCS values alone are not sufficient for the unequivocal assignment of *E*/*Z*‐isomers. They rather provide a reproducible and additional parameter that, when combined with retention behavior, UV absorption, and HR‐MS/MS spectra, significantly increases confidence in structural assignments.

Firstly, we observed that diastereomers, particularly *E/Z*‐isomers, exhibited differences in CCS values despite identical *m/z*. For instance, (*Z*)‐resveratrol (CCS 154.8 Å^2^, CV 0.01%) displayed lower mobility than (*E*)‐resveratrol (CCS 157.5 Å^2^, CV 0.08%), reaching statistical significance at *p* < 0.05 (*n* = 3). Representative ESI(−)‐HR‐MS/MS spectra and EIMs of (*E*)‐ and (*Z*)‐resveratrol are exemplarily provided in Figures S1 and S2. A similar discrepancy of 2.6 Å^2^ was noted between (*Z*)‐piceatannol (CCS 156.9 Å^2^) and (*E*)‐piceatannol (CCS 159.5 Å^2^). These findings align with recent studies reporting smaller CCS values for (*Z*)‐ compared to (*E*)‐isomers of various phenolic compounds, such as hydroxycinnamic acids and acylated anthocyanins carrying them as acyl moieties, underscoring the potential of ion mobility spectrometry in distinguishing *E*/*Z*‐isomers [15, 45].

Interestingly, in our study, this trend was predominantly evident for monomeric stilbenoids. CCS of dimeric stilbenoid diastereomers showed negligible differences, as demonstrated by nearly identical values of the tentative (*Z*)‐ɛ‐viniferin (CCS 206.4 Å^2^) and the unambiguously identified (*E*)‐ɛ‐viniferin (CCS 206.3 Å^2^). The precision of our CCS determination amounted to ±0.2 Å^2^, as recently reported by our working group [15].

Nevertheless, for tetrameric stilbenoids, an opposite trend was observed with the (*Z*)‐isomers exhibiting slightly higher CCS values than their (*E*)‐counterparts. For example, (*Z*)‐r2‐viniferin (#H9) showed a CCS of 289.3 Å^2^, compared to 285.1 to 287.9 Å^2^ for its (*E*)‐isomer (#H0/H10). This reversed IMS separation pattern for *cis*‐*trans* isomers has previously been reported for certain positional isomers of caffeoylquinic acids [46].

Nonetheless, we consistently observed that analyses of (*E*)‐stilbenoids generally featured one signal in the mobilogram when using our instrument settings, while the respective (*Z*)‐isomer additionally featured a second signal matching the one of the (*E*)‐isomer (data not shown in Table 1). The presence of two signals for (*Z*)‐isomers indicates that they may convert more readily into the (*E*)‐form than vice versa. The conversion is thought to occur during or after the ionization process in the source or within the TIMS cell. This phenomenon of “in‐source” isomerization has recently also been described by Schnitker et al. (2024) for acylated anthocyanins carrying (*Z*)‐hydroxycinnamoyl moieties [15].

Furthermore, we commonly observed additional signals, e.g., corresponding to formic acid (FA) adducts ([M–H+FA]^−^) or putative dimeric ions ([2 M–H]^−^) formed during or after ionization and during the trapping cycle. For instance, even in the nonirradiated sample, (*E*)‐resveratrol exhibited its characteristic CCS of 157.5 Å^2^, along with an additional signal at 206.3 Å^2^ ([2 M–H]^−^ also releasing [M–H]^−^ detected in the respective extracted ion mobilograms), suggesting the presence of a stilbenoid dimer like ɛ‐viniferin (data not listed in Table 1). This effect is illustrated in Figure 2B (heatmap of the untreated grape cane extract), where a signal at the mobility (1/K_0_) of 0.732 Vs/cm^2^ (#10, CCS = 157.7 Å^2^), along with a second signal at 1/K_0_ of 1.000 Vs/cm^2^ (CCS = 206.4 Å^2^).

Formic acid adducts were particularly observed for dimeric stilbenoids. For instance, the untreated (*E*)‐ε‐viniferin sample exhibited two different mobilities (Table 1, #F0) for the precursor ions with C_28_H_21_O_6_^−^ (calc. *m/z* 453.1344), one of which was attributed to the in‐source‐formed formic acid (CH_2_O_2_) adduct with C_29_H_23_O_8_^−^ (calc. *m/z* 499.1398). To obtain the correct mobility value for (*E*)‐ε‐viniferin, the extracted ion mobilogram (EIM) of the [M–H+FA]^−^ adduct was overlaid with that of the [M–H]^−^ ion. The comparison revealed that one of the mobility features appeared at the same mobility in both traces (219.6 Å), indicating it originated from the [M–H+FA]^−^ adduct, while the other (206.4 Å) was exclusive to the [M–H]^−^ trace, thus representing the true mobility of (*E*)‐ε‐viniferin and enabling the distinction between the actual analyte and its adduct. In the grape cane extract, this effect of overlapping mobility features for [M–H]^−^ ions and their corresponding [M–H+FA]^−^ adducts further hindered the precise assignment of specific CCS values for the tentatively identified stilbenoid dimers (Table 2, #11 and #12), thus limiting the reported CCS to those for the corresponding formic acid adducts as only one mobility was determined. Notably, aqueous formic acid was used as mobile phase A in this study to optimize the chromatographic separation. Conceivably, the formation of these adducts is due to the interaction of formic acid with the compounds during the ionization process.

## 4 Conclusion

Our examination of individual authentic stilbenoids and stilbenoid‐rich grape cane extracts by HPLC‐DAD‐ESI(−)‐TIMS‐QTOF‐HR‐MS/MS has yielded new insights into structural features and photodegradation pathways of these compounds. HR‐MS/MS spectra were instrumental for the identification of known compounds and additionally supported tentative structure annotation of novel degradation products. While TIMS only partly supported the structural elucidation, it proved particularly useful for differentiating isomeric structures, such as (*E*)‐ and (*Z*)‐stilbenoids, through the evaluation of full ion mobilograms rather than single CCS values. Although CCS values largely reflected molecular mass, subtle yet reproducible differences suggested further potential for structural interpretation beyond mass‐based trends.

In addition to the capabilities of ion mobility, existing knowledge on resveratrol photodegradation proved instrumental in identifying characteristic breakdown products of more complex stilbenoids. In particular, the consistent formation of hydroxybenzaldehydes could be of future relevance, especially for applications involving light exposure, such as in cosmetic or biopesticide formulations. To further broaden the analytical scope, future investigations might also include the positive ion mode, which could offer complementary insights into ionization behavior and structural annotation of stilbenoid derivatives.

## Author Contributions

**Paul Besrukow:** conceptualization, methodology, investigation, validation, data curation, project administration, writing – original draft, writing – review and editing, formal analysis, resources, visualization. **Friederike A. Schnitker:** writing – review and editing, validation, formal analysis, data curation. **Ralf Schweiggert:** supervision, data curation, conceptualization, writing – review and editing, funding acquisition, project administration, resources. **Christof B. Steingass:** conceptualization, methodology, investigation, validation, data curation, writing – review and editing, formal analysis, visualization.

## Funding

This work was supported by the German Research Foundation (DFG, project no. INST 370/1–1 FUGG). The project was partly supported by funds of the Federal Ministry of Food and Agriculture (BMEL) based on a decision of the Parliament of the Federal Republic of Germany, via the Federal Office for Agriculture and Food (BLE) under the Federal Program for Ecological Farming and Other Forms of Sustainable Agriculture in the scope of the joint project “VITIFIT” (2818OE004).

## Conflicts of Interest

The authors declare no conflicts of interest.

## Supporting information


**Figure S1:** HPLC‐DAD and ESI(−)‐HR‐QTOF‐MS extracted ion current (EIC) chromatograms of *m/z* 227.0714 resembling deprotonated resveratrol (A), UV absorption spectra of (*E*)‐ and (*Z*)‐resveratrol (B), ESI(−)‐HR‐QTOF‐MS/MS spectra (C) and a tentative mass fragmentation pathway (D) of resveratrol own illustration, modified according to Moss et al. (2013).
**Figure S2:** Exemplarily extracted ion mobilograms (EIMs) of m/z 227.0714 representing the deprotonated molecules [M−H]− of (E)‐ and (Z)‐resveratrol.
**Table S1:** HPLC‐DAD‐ESI(−)‐TIMS*‐*QTOF‐HR‐MS/MS data of additional authentic standard solutions.
