# Supplementary material for: Characterization of Grape Cane Stilbenoids and Their Photo‐Degradation Products by HPLC‐DAD Coupled With Trapped Ion Mobility Spectrometry and High‐Resolution Tandem Mass Spectrometry
Source: Rapid Commun Mass Spectrom. 2026 May 5;40:e70074. doi: 10.1002/rcm.70074 (PMC13145325; doi:10.1002/rcm.70074)
Supplement: Supplementary file 1 — Figure S1: HPLC‐DAD and ESI(−)‐HR‐QTOF‐MS extracted ion current (EIC) chromatograms of m/z 227.0714 resembling deprotonated resveratrol (A), UV absorption spectra of (E)‐ and (Z)‐resveratrol (B), ESI(−)‐HR‐QTOF‐MS/MS spectra (C) and a tentative mass fragmentation pathway (D) of resveratrol own illustration, modified according to Moss et al. (2013). Figure S2: Exemplarily extracted ion mobilograms (EIMs) of m/z 227.0714 representing the deprotonated molecules [M−H]− of (E)‐ and (Z)‐resveratrol. Table S1: HPLC‐DAD‐ESI(−)‐TIMS‐QTOF‐HR‐MS/MS data of additional authentic standard solutions. [file RCM-40-e70074-s001.docx]

**Characterization of grape cane stilbenoids and their photo-degradation products by HPLC-DAD coupled with trapped ion mobility spectrometry and high-resolution tandem mass spectrometry**

**Supporting Information**

Paul Besrukow^1,*^, Friederike A. Schnitker^1^, Ralf Schweiggert^1^ , Christof B. Steingass^1^

^1^Department of Beverage Research, Chair of Analysis and Technology of Plant-based Foods, Geisenheim University, 65366 Geisenheim, Germany

*Corresponding author: paul.besrukow@hs-gm.de

**Table S1:** HPLC-DAD-ESI(–)-TIMS*-*QTOF-HR-MS/MS data of additional authentic standard solutions.

| **Compound** | *t*_R_ (min) | λ_max_  (nm) | Exp. [M–H]^–^  (*m/z*) | Calc. [M–H]^–^  (*m/z*) | Error  (ppm) | Sum  formula | ESI(–)-HR-MS/MS (*m/z* (% base peak intensity) | ^TIMS^CCS_N2_  (Å^2^) ± CV (%) | Mobility  1/K_0_  (Vs/cm^2^) |
| --- | --- | --- | --- | --- | --- | --- | --- | --- | --- |
|  |  |  |  |  |  |  |  |  |  |
| 3,5-dihydroxybenzoic acid | 2.5 | 249, 307 | 153.0195 | 153.0193 | –1.1 | C_7_H_5_O_4_^–^ | 109.0290 (48), 67.0188 (52), 65.0032 (16), 41.0034 (100) | 123.5 ± 0.36 | 0.567 |
| 3,4-dihydroxybenzoic acid | 2.5 | 259, 293 | 153.0194 | 153.0193 | –1.1 | C_7_H_5_O_4_^–^ | 109.0298 (100), 108.0214 (28), 91.0187 (6), 65.0033 (6), 41.0034 (4) | 121.2 ± 0.05 | 0.557 |
| 3,5-dihydroxybenzaldehyde | 2.9 | 269, 329 | 137.0246 | 137.0244 | –1.6 | C_7_H_5_O_3_^–^ | 95.0140 (37), 93.0351 (94), 65.0039 (13), 41.0039 (100) | 120.3 ± 0.22 | 0.547 |
| 3,4-dihydroxybenzaldehyde | 3.0 | 279, 311 | 137.0245 | 137.0244 | –0.7 | C_7_H_5_O_3_^–^ | 108.0215 (12), 91.0191 (3), 81.0347 (2), 65.0037 (4), 41.0038 (3) | 118.1 ± 0.17 | 0.537 |
| 4-hydroxybenzoic acid | 3.0 | 254 | 137.0244 | 137.0244 | –0.2 | C_7_H_5_O_3_^–^ | 109.0303 (5), 108.0255 (7), 93.0322 (2), 92.0263 (2), 91.0192 (4), 41.0041 (3) | 119.4 ± 0.75 | 0.543 |
| 4-hydroxybenzaldehyde | 3.8 | 284 | 121.0298 | 121.0295 | –2.1 | C_7_H_5_O_2_^–^ | 92.0272 (30), 41.0039 (3) | 116.4 ± 0.09 | 0.524 |

*t*_R_: retention time, λ_max_: UV/Vis absorption maxima, CCS: collision cross section, CV: coefficient of variation (*n* = 3).


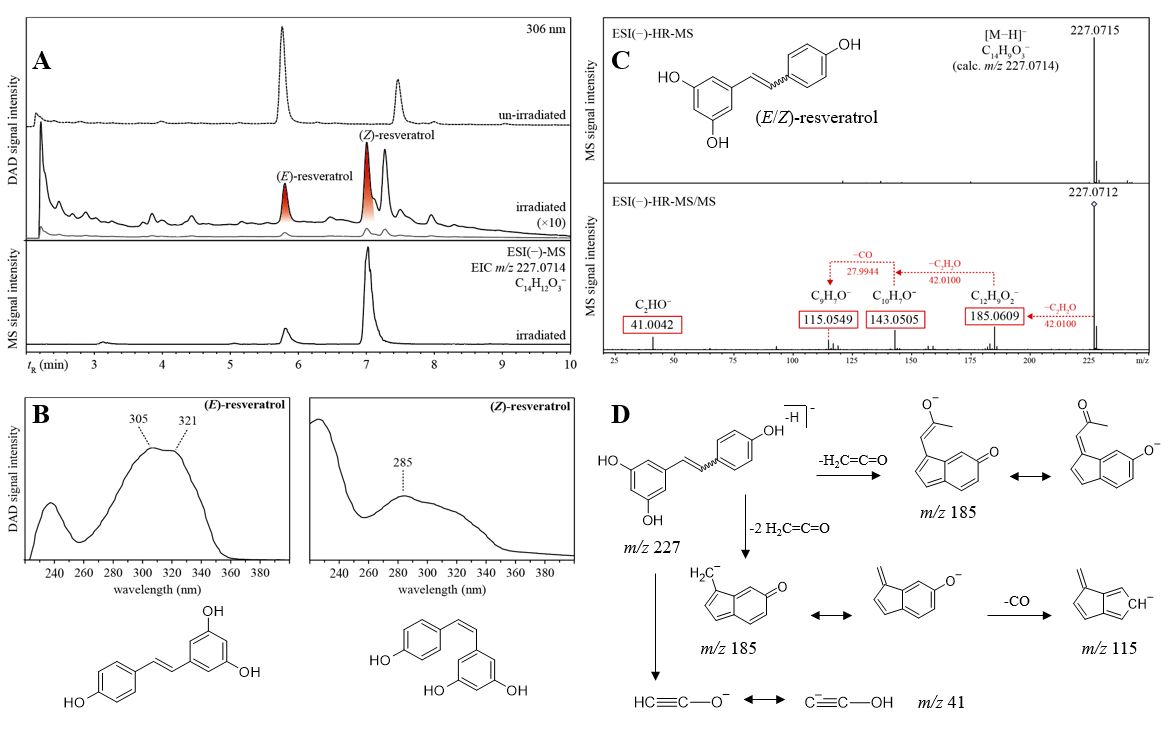


Figure S1: HPLC-DAD and ESI(−)-HR-QTOF-MS extracted ion current (EIC) chromatograms of *m/z* 227.0714 resembling deprotonated resveratrol (A), UV absorption spectra of (*E*)- and (*Z*)-resveratrol (B), ESI(−)-HR-QTOF-MS/MS spectra (C) and a tentative mass fragmentation pathway (D) of resveratrol (own illustration, modified according to Moss et al. (2013).


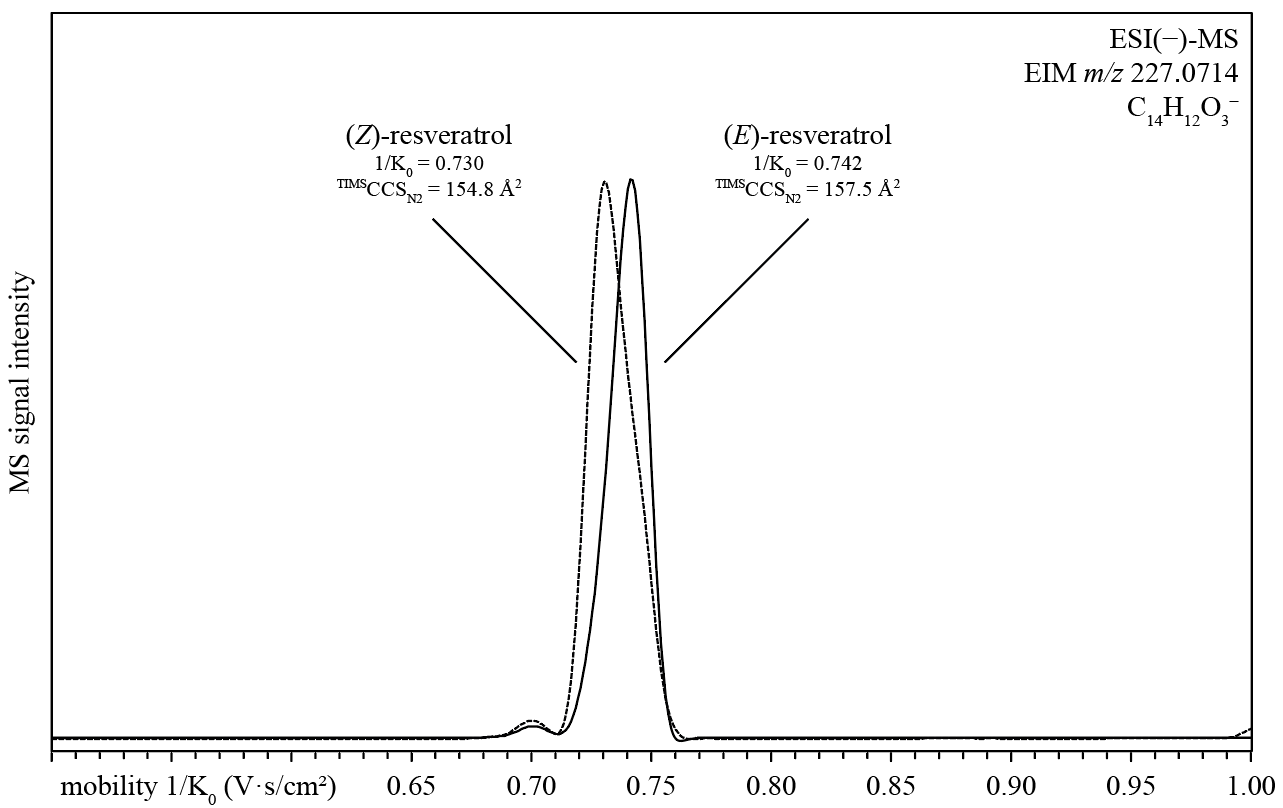


Figure S2: Exemplarily extracted ion mobilograms (EIMs) of *m/z* 227.0714 representing the deprotonated molecules [M−H]^−^ of (*E*)- and (*Z*)-resveratrol.

**References used in the Supporting Information**

Moss R, Mao Q, Taylor D, Saucier C. Investigation of monomeric and oligomeric wine stilbenoids in red wines by ultra-high-performance liquid chromatography/electrospray ionization quadrupole time-of-flight mass spectrometry. *Rapid Commun Mass Spectrom*. 2013;27(16):1815-1827. doi:10.1002/rcm.6636.
